# Discoloration of Historical Plastic Objects: New Insight into the Degradation of *β*-Naphthol Pigment Lakes

**DOI:** 10.3390/polym13142278

**Published:** 2021-07-12

**Authors:** Anna Micheluz, Eva Mariasole Angelin, João Almeida Lopes, Maria João Melo, Marisa Pamplona

**Affiliations:** 1 Conservation Science Department, Deutsches Museum, Museumsinsel 1, 80538 Munich, Germany; a.micheluz@deutsches-museum.de; 2 Department of Conservation and Restoration and LAQV-REQUIMTE, NOVA School of Science and Technology, NOVA University Lisbon, 2829-516 Lisbon, Portugal; mjm@fct.unl.pt; 3 iMed.ULisboa-Research Institute for Medicines, Faculty of Pharmacy, University of Lisbon, Av. Prof. Gama Pinto, 1649-003 Lisbon, Portugal; jlopes@ff.ulisboa.pt

**Keywords:** historical plastic objects, *β*-naphthol pigment lakes, discoloration, fading, colorant degradation, photodegradation, ATR-FTIR, EGA-MS, TD-GC/MS, Py-GC/MS

## Abstract

Light is a determining factor in the discoloration of plastics, and photodegradation processes can affect the molecular structures of both the polymer and colorants. Limited studies focused on the discoloration of heritage plastics in conservation science. This work investigated the discoloration of red historical polyethylene (PE) objects colored with PR 48:2 and PR 53:1. High-density and low-density PE reference polymers, neat pigment powders, and historical samples were assessed before and after accelerated photoaging. The applied methodology provided insight into the individual light-susceptibility of polyethylenes, organic pigment lakes, and their combined effect in the photoaging of historical plastic formulations. After light exposure, both PE references and historical samples yellowed, PR53:1 faded, and PR 48:2 darkened; however, both organic pigments faded severely in the historical samples. This highlights the role played by the plastic binder likely facilitating the pigment photofading. Fourier transform infrared spectroscopy and mass spectrometry techniques—EGA-MS, PY-GC/MS, and TD-GC/MS—were successfully employed for characterizing the plastic formulations and degradation. The identification of phthalic compounds in both aged *β*-naphthol powders opens new venues for studies on their degradation. This work’s approach and analytical methods in studying the discoloration of historical plastics are novel, proving their efficacy, reliability, and potentiality.

## 1. Introduction

Museums are traditionally dedicated to collecting, investigating, preserving, and exhibiting testimonies from the past and portraying memories associated with their role and impact, among others, in the social context. The development of man-made polymers from the mid-19th century significantly changed the modern world. Those changes cover all the aspects of society, including the lifestyle. Therefore, polymer-based objects are now increasingly common in heritage collections as testimonies of our past. Today, plastic objects are found in every type of museum worldwide, ranging from designer pieces and unique and valuable artworks to mass-produced objects, partially or entirely made of plastic.

Since the first Parkesine plastics became popular [1], semi-synthetic and synthetic plastic objects have been made in all different shapes, sizes, and colors, thanks to the versatility of these materials and the manufacturing development from the early 20th century onwards. Between the visible characteristics, color is the property that made plastics so attractive, especially in modern plastic-based objects generally characterized by vibrant and colorful surfaces [2].

Additives are often mixed/blended with the basic polymer in the plastic formulation. The addition of these chemicals (e.g., plasticizers, flame retardants, antioxidants, acid scavengers, light and heat stabilizers, lubricants, antistatic agents, colorants, etc.) in plastic manufacturing improves the processing, end-use performance, and aging properties of the polymer. Since most plastic resins are weakly colored (white-to-pale yellow) or colorless, colorants can be added to the polymer resin in the manufacturing process to internally color the formulation and impart a visible color to the plastic objects [3,4,5]. This will influence the aesthetics of plastic objects, such as the color appearance, turning a commodity plastic into a more visually pleasing product [6,7,8].

In the world of plastics, hundreds of synthetic colorants appear in the colorists’ palette. Following the literature of plastic coloring technology [3,4,5], colorants are used in very small concentrations (0.1–5%), in the form of soluble organic dyes or as finely divided insoluble organic/inorganic pigments. They offer a wide range of colors for standard coloration, and the use of specific colorant types (e.g., metallic, pearlescent, fluorescent, phosphorescent, thermochromic, and photochromic) can impart a “special effects” appearance. Colorants can be used in pure tone or mixed to achieve the desired color appearance. Often additives such as fillers and extenders are incorporated into the plastic resin, together with the colorants, to improve processing, physical and mechanical properties, or to reduce cost.

Plastics in collections are challenging to preserve, as they tend to degrade faster than other materials found in artworks [9,10,11,12,13,14,15,16,17]. Synthetic organic/inorganic pigments also have stability issues [18,19,20,21,22,23,24,25] and their alteration in modern polymeric paints continues to be a subject of ongoing research in art conservation [26,27,28,29,30,31]. Both polymers and pigments are known to be sensitive towards aging, which can lead to color change.

Discoloration (alteration of original color) of plastic materials dramatically affects the perception of a wide range of polymer-based objects [1,32,33], making its study a research priority in conservation science of cultural heritage. When a color change is visible in aged plastic, a chemical reaction has already taken place. Discoloration is usually a symptom of photo-induced damage, where light caused chemical changes at the molecular level in the polymer matrix and/or colorants. Free radicals and atmospheric oxygen can also be involved in the chemical reactions.

The photodegradation of polymer resins and its contribution to discoloration (yellowing and darkening) has been widely studied [34,35,36,37,38], while only recently, the fading of organic pigments has been evaluated in conservation science, focusing mainly on the identification of the faded organic pigments in historical plastic objects [39,40,41]. Although few key research studies from the polymer science community discussed the degradation of pigmented polymers [42,43,44], they were not comprehensive enough in describing the photo-fading mechanisms of the organic pigments.

The study of the organic pigments and their fading phenomena in historical plastics represents a methodological and analytical challenge, firstly because of technical difficulties in their identification [41], secondly due to the complexity of their degradation pathways [43], and thirdly due to the often-unknown exposure (duration and intensity) to the agents of degradation (environmental factors). Once the decay mechanism starts, it is usually irreversible, and the pigment modification results in a chemical change of the molecule’s pigment structure that causes a color shift or loss. The light-resistance of organic pigments is always related to the whole plastic system. Therefore, besides the intrinsic fastness properties of the pigment, the properties of polymer and additives can affect the organic pigments’ light stability. Polymers have different photo-reactivity depending on their chemistry and structure. Their role is key in pigment photodegradation as they constitute the major component of the pigment environment. Indeed, the polymeric matrix can trigger or be part of the chemical reactions that cause the pigment degradation. So far, most of the studies about the interaction between polymers and synthetic organic pigments in heritage science were focused on the conservation of modern polymeric paints with synthetic binders [45,46,47,48,49,50,51,52]. Additives play a two-fold role in the photo-fading mechanism: (i) on one hand, chemicals such as light stabilizers and antioxidants improve the color stability and prolong the objects’ longevity by protecting the plastic against harmful environmental factors (e.g., light and oxygen); (ii) on the other hand, the chemistry of all possible interactions in the triad polymer-pigment-additives has not been fully understood and so estimations of the process outcome are difficult.

*β*-naphthol pigment reds, substituted 1-arylhydrazone-2-naphthols, are the most extensive family of organic red colorants [53]. Their impact is well-documented in historical sources [54,55,56,57]. They were one of the first pigment categories used in the coloring of plastics [54,55] and the subject of relevant publications [55,58,59,60,61,62,63,64,65,66,67]. In historical collections, problems related to their fading in printing inks [68], modern and contemporary paintings [69], as well as plastic artifacts [39,41] are well-documented. Nevertheless, little is known about their photochemistry in cultural heritage collections because few studies have focused on their degradation [70,71,72].

C.I. Pigment Red (PR) 48 (C.I. no. 15865) and C.I. PR 53 (C.I. no. 15585) pigment-type lakes were highlighted as photosensitive in polyolefin plastics [39,41]. However, their fading has not been studied yet, nor the influence of the formulation components on their degradation.

In this work, the discoloration of red historical polyethylene (PE) objects colored with PR 48:2 and PR 53:1 was investigated by a novel stepwise approach. The aim was to characterize the light-induced alterations of the polymer and organic pigments. To this end, the analytical strategy was planned to firstly identify the formulations’ constituents of the historical samples, secondly analyze separately the discoloration of the polymer matrix and *β*-naphthol pigment lakes due to photodegradation, and thirdly observe their combined effect on a plastic formulation. As PE can display different amounts of chain branching, high-density (HD) and low-density (LD) PE polymer references were considered. PR 48:2 and PR 53:1 in powder form served as references for the pigments in the study. Formulations of PE with organic pigments were chosen to be taken from real historical objects, although being naturally aged to an unknown extent, because they were industrially produced with methods from the past, likely similar to those implemented in other cultural objects. Within the timeframe of this study, unaged reference formulations reproducing historical production methods were not achievable, remaining therefore a topic for future research. Samples were assessed before and after photoaging experiments by means of Fourier transform infrared spectroscopy (FTIR) and mass spectrometry (MS) techniques. Colorimetric measures and microscope observations were made to characterize the color changes. An accelerated aging test was used to simulate the discoloration process. As wavelengths no longer than ca. 290 nm reach the earth’s surface, radiation at λ ≥ 300 nm would probably be absorbed by the chromophores present in the plastics, reproducing a real exposure scenario. The PE polymer was selected as its photochemistry is well-known [73,74] and because *β*-naphthol pigments were widely used in the past to color polyolefins [3,4,5,54]. This study also led to exploring the applicability and effectiveness of MS-based techniques in the molecular characterization and degradation assessment of both polymer and organic pigments. To these ends, the performance of evolved gas analysis-mass spectroscopy (EGA-MS) and thermal desorption-gas chromatography/mass spectrometry (TD-GC/MS), combined with pyrolysis-gas chromatography/mass spectrometry (Py-GC/MS), were tested. Unsupervised modeling principal component analysis (PCA) was applied to help with the interpretation of multivariate thermographic datasets.

## 2. Materials and Methods

### 2.1. Samples

**Figure 1:**
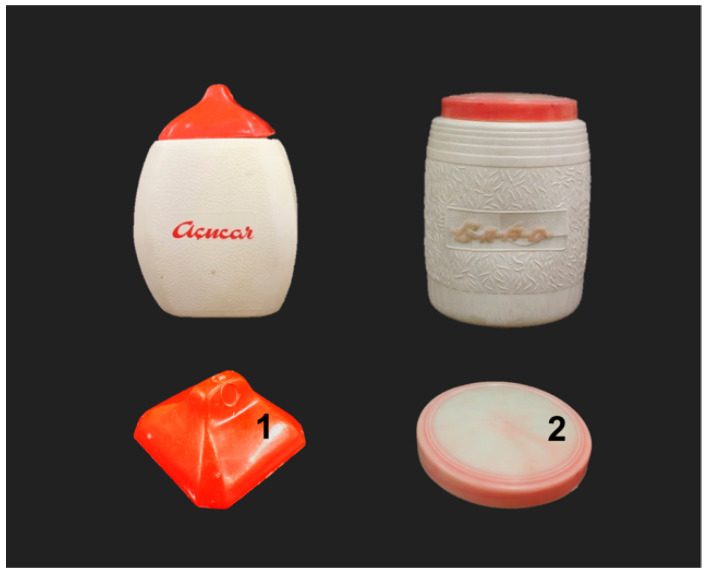
The historical plastic objects under study, lid 1 made of PE with PR 48:2 (**bottom left**), lid 2 made of PE with PR 53:1 (**bottom right**).

Historical plastic objects: The selection includes two food containers with a white body and red lid from the 1950s–1970s, gathered from a Portuguese private collection. One container has a printed red lettering inscription reading “*Açucar*” (“sugar”, all the translations in the paper were made by the authors) and the other a three-dimensional (3D) red lettering inscription reading “*GRÃO*” (“chickpea”) (Figure 1, top). No color alterations were observed for lid 1, while lid 2 showed an inhomogeneous color as a consequence of discoloration (Figure 1, bottom). In a previous study, Angelin et al. [41] characterized lid 1 and lid 2 as made of PE, and identified PR 48 and PR 53 as the main red coloring agents of lids 1 and 2, respectively. Further results on their elemental and molecular characterization are summarized in Supplementary Table S1 [41]. Both PR 48 and PR 53 pigment-type lakes can be complexed by more than one cation type [53]. Since only Ca was detected as a possible counter ion in lid 1, the presence of calcium salt type PR 48:2 is suggested. Lid 2 is most likely colored with the barium PR 53:1 type, considering that all the other salts do not or have little value in plastic applications [4,5].

HDPE and LDPE Polymers: Polymer reference samples (unaged) were obtained from non-colored and additive-free formulations of high-density polyethylene (HDPE) and low-density polyethylene (LDPE) (Repsol Polímeros, Sines, Portugal). With a JCS Shinha press and mold plates, pellets of HDPE and LDPE were molded into circular disks of 2.5 cm diameter by the application of heat (160 °C) and pressure (400 psi) for 3 to 5 min. Samples of ca. 1 mm of thickness were obtained. Samples No. 25 (HDPE) and 24 (LDPE) from the ResinKit^TM^ collection [33] also served as reference materials. In this study, these samples were exclusively used to facilitate interpretation of the PCA results.

Synthetic organic pigments: PR 48:2 and PR 53:1 (Clariant, Muttenz, Switzerland) pigments were used as reference pigment powders without purification or recrystallization. Molecular structures of the red synthetic organic pigments are reported in Supplementary Table S2.

### 2.2. Artificial Aging

Accelerating aging with polychromatic irradiation was carried out in a CO.FO.ME.GRA apparatus (SolarBox 3000e) (Milan, Italy) equipped with a Xenon-arc light source (λ ≥ 300 nm) with constant irradiation of 800 W/m^2^. The temperature inside the apparatus was maintained at approximately 40 °C. The experimental setup for artificial aging is shown in Supplementary Figure S1. The reference and historical plastic samples were cut in two different sizes (ca. 5 × 5 × 1 mm^3^, and ca. 12 × 12 × 1 mm^3^), then placed in 10 × 10 quartz cuvettes (type-110 QS, cutoff λ = 190 nm, Hellma GmbH, Müllheim, Deutschland) and under a Pyrex glass (cutoff λ = 280 nm). The quartz cells were not closed with a cap during the aging experiment and, after monitoring measurements, the same surface of the samples was exposed to irradiation. Approximately 10 mg of organic pigment powders were placed onto a glass slide (single concavity) and covered with a Pyrex glass. Samples were irradiated for 110 h (t_1_), 220 h (t_2_), 550 h (t_3_), and 770 h (t_4_). Another set of samples was kept in the dark for control, 0 h (t_0_). Historical lids and polymer reference samples were considered homogenous, having a uniformly dispersed composition of the plastic material. During the aging experiment, the historical lids (ca. 12 × 12 × 1 mm^3^) were analyzed by ATR-FTIR. At each irradiation time, one sample (ca. 5 × 5 × 1 mm^3^) for each lid was taken and analyzed by EGA-MS and Py-GC/MS. To compare the results of the aged historical samples, ATR-FTIR and EGA-MS analyses were carried out on the HDPE and LDPE samples following the same experimental procedure in order to characterize the photo-induced molecular changes of the polymer references. All samples were observed under the stereomicroscope and color variation of the plastic samples was monitored by colorimetry. Organic pigment powders were characterized before (t_0_) and after (t_4_) artificial aging by EGA-MS and Py-GC/MS. Sampling of the samples at the uppermost layer of the exposed surface was performed for MS measurements.

### 2.3. Attenuated Total Reflection Fourier-Transform Infrared Spectroscopy (ATR-FTIR)

The exposed surfaces of the historical and polymer reference samples were characterized by IR spectroscopy in attenuated total reflection (ATR-FTIR) with the Handheld 4300 FTIR spectrophotometer (Agilent Technologies Inc., Santa Clara, CA, USA), equipped with a ZnSe beam splitter, a Michelson interferometer, and a thermoelectrically cooled DTGS detector. Spectra were acquired with a diamond ATR module, 128 scans, and 4 cm^−1^ resolution, between 4000 and 650 cm^−1^. This ATR module allows the analysis of samples with a minimum size of 200 µm. Background spectra were collected between every acquisition. The OriginPro 8 (OriginLab Corportation, Northampton, MA, USA) software was used to analyze the spectra, which are shown as acquired, without baseline corrections or normalizations. The same spot of the plastic surface was analyzed at each of the artificial aging intervals to guarantee monitoring accuracy.

### 2.4. Color Measurements

A Microflash mobile colorimeter (DataColor International, Lucerne, Switzerland) was employed for measuring and monitoring the color of the historical and polymer reference samples during the artificial aging. The colorimeter was equipped with a Xenon lamp. The 1976 CIELAB color coordinates (L*, a*, b*) were calculated over an 8 mm diameter measuring area, considering the D65 standard illuminant and the 10° colorimetric observer (CIE 1964). The reflected specular component (SCE mode) was excluded from the measurements. The instrument was calibrated with a white (100% reflective) and black (0% reference) balance, in accordance with the DataColor calibration procedure. The white (porcelain) and black trap standards were provided by the manufacturer. Both the equipment measuring head and the sample were placed in a custom-made positioning mask, which allowed to obtain color measurements on the same area at each artificial aging interval. The reported values are the average of three measurements collected on the measurement area of the exposed plastic surfaces, which proved to be sufficient to guarantee reproducibility. Total color variation was calculated according to CIELAB 1976 (ΔE*_ab_) and CIEDE 2000 (Δ*E*_00_) expressions [75].

### 2.5. Optical Microscopy (OM)

OM was used to identify the color and distribution of the pigment particles in the historical plastic samples before and during artificial aging. Images were acquired using a Axioplan 2 Imaging system (HAL 100) (Zeiss, Oberkochen, Germany) coupled to a DXM1200F digital camera (Nikon Corporation, Tokyo, Japan) and ACT-1 software (Nikon Corportation, Tokyo, Japan). Dark-field illumination was used.

### 2.6. Stereomicroscope

All samples were observed before and at the different irradiation times under a MZ16 stereomicroscope (Leica Microsystems GmbH, Wetzlar, Germany) (7.1× to 115× zoom range), and images were acquired using a Leica ICD digital camera and a fiber-optic light Leica system (Leica KI 1500 LCD) coupled with the stereomicroscope.

### 2.7. Evolved Gas Analysis-Mass Spectroscopy (EGA-MS)

The exposed surfaces of the historical and polymer reference samples as well as the pigments in powder were analyzed by EGA-MS. Around 200 µg of each sample was added directly into an 80 µL stainless-steel Eco-cup sample holder (Frontier Laboratories Ltd., Koriyama, Japan) and analyzed by a Multi-shot Pyrolyzer EGA/PY-3030D (Frontier Laboratories Ltd.). The temperature program was set up from 100 to 700 °C (hold for 5 min) at a ramp of 20 °C/min. Evolved gas was transferred directly into the 5977B MSD mass spectrometer (Agilent Technologies Inc., Santa Clara, CA, USA) through a deactivated and uncoated stainless-steel transfer Frontier Ultra Alloy^®^ EGA tube (UADTM 2.5N-2.5m-I.D. 0.15 mm, O.D. 0.47 mm, Frontier Laboratories Ltd.) maintained at 300 °C in the 7890B GC system oven (Agilent Technologies Inc.), with helium as carrier gas at a flow rate of 1.2 mL/min and a split ratio of 25:1. Ion detection was carried out in the *m*/*z* range of 25–550. The thermogram interpretations and the volatile identifications were performed by comparison and selective *m*/*z* extraction with the software and database F-Search 3.5.0 (Frontier Laboratories Ltd.) and literature data [76]. At each irradiation interval, the EGA-MS analysis was performed twice for all samples.

### 2.8. Single-Shot Pyrolysis-Gas Chromatography/Mass Spectrometry (Py-GC/MS)

Pyrolysis was performed on the pigments’ powders for the characterization of their markers and their subsequent detection in the exposed surfaces of the historical PE lids. Around 100 µg of sample was placed into an 80 µL stainless-steel Eco-cup and pyrolyzed at 600 °C for 6 s using the same instrument described in the EGA-MS section. The pyrolysis interface was maintained at 300 °C. GC separations were performed using a Frontier UA5 capillary column (30m-0.25F, 30 m × 250 µm × 0.25 µm, Frontier Laboratories Ltd.), using helium as a carrier gas at flow rate of 1.2 °C/min and a split ratio of 15:1. The column temperature was programmed from 35 °C (hold for 1 min), increasing the rate at 16 °C/min to 220 °C, then at 10 °C/min to 315 °C (hold for 2 min). The MS parameters were: electron impact ionization (EI, 70 eV) in positive mode, transfer line at 280 °C, ion source at 230 °C, quadrupole at 150 °C, and scanning mass range 20–600 *m*/*z*. Data interpretation was performed by MassHunter Workstation Ver. B.0700 SP2 (Agilent Technologies Inc.) software and compound identification was accomplished by interpretation of their EI mass spectra, in comparison to NIST MS Search 2.2, F-Search 3.5.0 (Frontier Laboratories Ltd.) databases and literature data [39,76,77].

### 2.9. Thermal Desorption-Gas Chromatography/Mass Spectrometry (TD-GC/MS)

The thermal desorption analysis was performed on the basis of the previous EGA-MS results, analyzing the exposed surfaces of the historical polymeric matrices, focusing only on the volatile fractions for the additive’s characterization. Around 200 µg of sample was added directly into an 80 µL stainless-steel Eco-cup sample to be analyzed with the same instrument described in the previous EGA-MS section. The temperature program started from 50 °C (hold for 30 s) to 320 °C (hold for 3 min), with an increasing ratio of 20 °C/min. The volatile focalization was obtained by a cryo-trap at –180 °C at the beginning of the GC column. GC separations were performed using a Frontier UA5 capillary column (see previous section), with helium as a carrier gas at a flow rate of 1.2 mL/min and a split ratio of 15:1. The injector temperature was set at 300 °C. The column temperature program ran from 40 °C (hold for 2 min), increasing at a rate of 20 °C/min until 280 °C (hold for 15 min). The MS parameters were: EI (70 eV) in positive mode, transfer line at 280 °C, ion source at 230 °C, quadrupole at 150 °C, and scanning mass range 29–550 *m*/*z*. Data interpretation was performed by MassHunter Workstation Ver. B.0700 SP2 (Agilent Technologies Inc.) software and compound identification was accomplished by interpretation of their EI mass spectra, in comparison to NIST MS Search 2.2, F-Search 3.5.0 (Frontier Laboratories Ltd.) databases and literature data [76].

### 2.10. Chemometric Method

The PCA method was employed to analyze the EGA thermograms, namely, to recognize differences at a multivariate level between the high-density and low-density PE polymers, as well as to identify similarities with the historical samples. The PCA was performed on the EGA curves collected from unaged and artificially aged HDPE and LDPE polymer references, as well as historical lids. Sets of two replicas for each sample at the different irradiation doses were considered in the PCA model. The mass spectrometric data were excluded from the PCA. The first test of the PCA (Method A) included EGA thermograms preprocessed with normalization by unit area. The second test (Method B) was performed on a dataset of descriptors generated directly from EGA results, including signal area, peak maximum time, maximum and minimum peak slopes, and peak width at half height. For method A, the dataset was mean-centered before the application of PCA. For Method B, autoscaling was used. Matlab R2016b (Mathworks, Natick, MA, USA) and the PLS toolbox version 8.2.1 (Eigenvector, Manson, WA, USA) were used for PCA modeling. To address the robustness of the PCA calibration, reference ResinKit^TM^ samples No. 24 and 25 were considered.

## 3. Results

### 3.1. Colorimetric Measures and Microscope Observations

After 770 h of irradiation, both PE reference samples and historical lids showed significant changes in color, surface texture, and cohesion. In accordance with the colorimetric results in Supplementary Figure S2, yellowing was observed in HDPE and LDPE samples, while the historical lids faded considerably. Indeed, all ΔE*_ab_ values indicate a visible color change (ΔE*_ab_ > 2.3 [78]), with the highest difference for the historical lids 1 and 2 (Supplementary Table S3). A detailed description of the colorimetric measurements is available in the Supplementary Material. Besides color change, both HDPE and LDPE samples cracked all over the exposed surfaces, as visible under the stereomicroscope (Supplementary Figure S3). On the other hand, the historical lids became brittle and broken with a network of tiny cracks on the top surface and alongside their entire thickness (Supplementary Figures S3 and S4), suggesting the occurrence of important light-induced chemical and physical modifications on the polymer molecular structure. Observing the side view of the lids’ samples (thickness ca. 1 mm, Supplementary Figure S4), it can be inferred that fading took place at the surface of the plastic samples. A color gradient between the uppermost layers (whitish) and the middle-bottom areas (reddish) of the samples is clearly visible. Pigment particles dispersed in the polymeric matrix are still visible under the optical microscope after 220 h of artificial aging in the faded sample from lid 2 (Supplementary Figure S5), although many particles entirely or partially lost their color.

A color change was also observed for both pigment powders. In the aged sample PR 53:1, the formation of a pinkish superficial layer was seen, while PR 48:2 became slightly darker after 770 h (Supplementary Figure S6).

### 3.2. Polymer Matrix: Reference Polymers and Historical Plastic Samples

ATR-FTIR and EGA-MS analyses were performed to characterize the polymer and its degradation. Particular attention was given to the identification of aging markers, which can be correlated to significant changes in the polymer structures and yellowing.

#### 3.2.1. ATR-FTIR Results

The ATR-FTIR spectra of the PE reference samples and historical lids presented the same main absorption bands (Supplementary Figure S7), with characteristic aliphatic stretching vibrations at 2916, 2848 cm^−1^, methylene-bending at 1472, 1463 cm^−1^, and methylene-rocking at 730, 719 cm^−1^ bands [79,80]. The two doublet bands correspond to the PE crystalline (1474 and 730 cm^−1^) and amorphous (1463 and 719 cm^−1^) contents [81].

Polyethylenes with different degrees of chain branching (i.e., HDPE and LDPE types [82]) can be distinguished by IR analysis. The presence of the band at ca. 1377 cm^−1^ can be used as a marker for LDPE identification as being specifically associated with the vibration of the –CH_3_ terminating groups of the short- and long-chain branching [83,84]. The ATR-FTIR spectra of the plastic samples prior to aging in the spectral range 1300–1430 cm^−1^ are depicted in Supplementary Figure S8. The PE reference samples match literature spectra [83,84] as respectively HD and LD. Lid 1 would be classified as HDPE, as previously suggested by Angelin et al. [41]. However, using the presence/absence of that marker for identifying the PE type of lid 2 could be misleading, because the plastic was naturally degraded, as observed by visual inspection and confirmed by the presence of bands at 1735 and 1714 cm^−1^ (Supplementary Figure S7), which indicate the formation of esters and carbonyl compounds (mainly carboxylic acids) respectively, characteristic of PE photo-oxidation [85]. This reflects the occurrence of the polymeric chain scission, which altered the degree of branching (extent of –CH_3_ terminating groups) of lid 2.

Yellowing (Supplementary Figure S2) and embrittlement (Supplementary Figure S3) are both characteristic symptoms of PE photo-oxidation [36,86,87,88]. The light-induced degradation of PE was identified by means of ATR-FTIR spectroscopy (Supplementary Figure S9) and involved the formation of oxidative products such as carboxylic acids (1714 cm^−1^), esters (1733 cm^−1^), lactones (1780 cm^−1^), ketones (1410 cm^−1^), vinyl (995 and 909 cm^−1^), and *t*-vinylene (965 cm^−1^) [85,89,90,91]. At ca. 1167 cm^−1^, a strong band was also observed, for which controversial assignments have been proposed, including branching [89,92], esters [90,93], and vinyl groups [94]. The same photo-oxidation products were identified in the ATR-FTIR spectra of reference and historical plastic samples.

After 770 h of irradiation, the accumulation of the carbonylic species, vinylenic and vinyl un-saturations, became more significant for LDPE reference and historical lid 2 samples (Supplementary Figure S9b,d). This indicates a higher degree of photo-oxidative degradation of both samples. LDPE is known to be more susceptible to undergo photo-oxidation reaction than HDPE due to the greater number of tertiary carbons present at the branch points [35,86], as observed experimentally in [95,96]. Before irradiation, lid 2 exhibited photo-oxidation already at early stages (Supplementary Figure S7) and, after 550 h of exposure, a broad and strong band centered at 1640 cm^−1^ also appeared, suggesting the formation of new unsaturated species (Supplementary Figure S9d) [85,89,90,91,92]. These structures are strong chromophores and, depending on the extent of the conjugated unsaturated system, they can be responsible for the deeper yellowing of lid 2 [36]. In the LDPE reference sample, this broad absorbance band is only hinted at (Supplementary Figure S9b).

Dramatic changes in the 1400–1300 cm^−1^ spectral region were also detected with aging (Supplementary Figure S10). After 110 h, scission of the HDPE reference polymer chain occurred, as indicated by the appearance of the –CH_3_ chain end groups (1374 cm^−1^). This increase of end methyl groups in HDPE due to thermo-photo-oxidation processes had already been observed and reported [89,90,92]. In contrast, the marker band for LDPE, at ca. 1377 cm^−1^, disappeared with aging. Both vibrational spectra of HDPE and LDPE reference samples were characterized by the emergence of a new band, at ca. 1360 cm^−1^, assigned to photo-induced un-saturations (ketone structures) [89].

#### 3.2.2. EGA-MS Results

**Figure 2:**
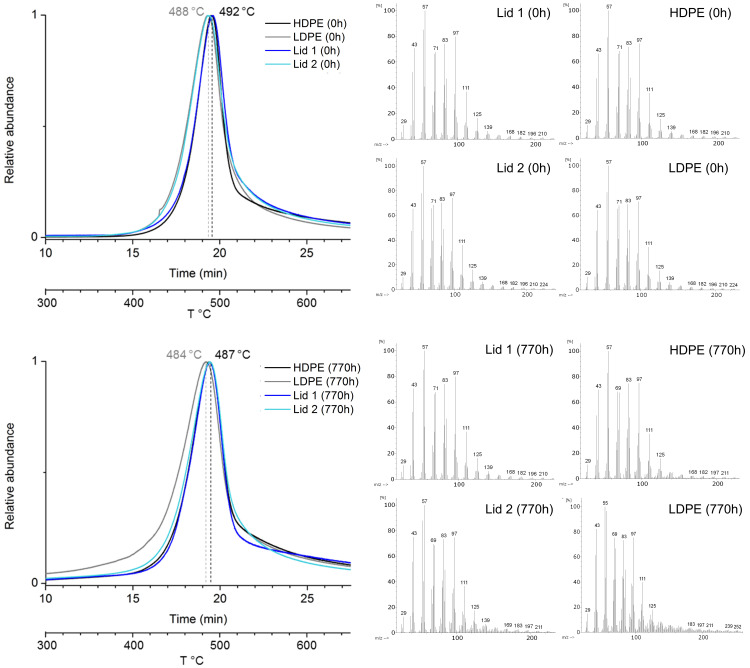
Comparison of the EGA chromatograms of the four PE samples at 0 h (**up**) and 770 h (**bottom**) with the related average mass spectra. On the top: at 0 h, the curves of lid 1 and HDPE overlay, the curves of lid 2 and LDPE overlay. On the bottom: at 770 h, the curves of both lids and HDPE overlay. All intensities are normalized.

The results of EGA-MS analysis confirmed that lids 1 and 2 are made of PE, with their average maximum peaks at 492 and at 488 °C, respectively (Figure 2).

In Figure 2, the thermograms of lids 1 and 2 are compared with LDPE and HDPE reference samples before and after aging. At 0 h, the EGA curves of lid 1 and HDPE were similar, with the maximum peak located at 492 °C and slightly narrower than the broader curves of lid 2 and LDPE, which have the maximum peak at 488 °C. Lid 1 is likely made of HDPE; however, since the sample of lid 2 was previously naturally aged, it is not possible to draw conclusions by comparing it with unaged PE reference samples. After aging (770 h), lid 1 and HDPE showed similar curves (their maximum decreased by 5 °C to 487 °C), the curve of lid 2 was kept almost constant, and the curve of the LDPE sample became even broader and shifted its peak to less than 4 °C (maximum at 484 °C). The distinction between unaged LDPE and HDPE by EGA-MS analysis is not simple because their temperature range of decomposition overlays (between 480 and 490 °C [97]) and the distinction between their maximums is 4 °C apart. However, the polymer nature of the historical lid 1 became recognizable after aging, as EGA-MS analysis was sensitive enough for detecting the higher susceptibility of LDPE to undergo photo-oxidation reaction than HDPE [35,86]. The shift to lower temperatures of the maximum of both EGA-curves after aging is associated with degradation—shorter polymer chains—which happens for both LDPE and HDPE. The broadening of the LDPE indicates a higher degree of degradation with the formation of various lengths of polymer chains. Thus, the HD nature of lid 1 can be suggested considering that it exhibits similar curves to those of HDPE. However, the curve of lid 2 remained almost constant (from 488 to 487 °C). Considering the maxima of the curves, it is not possible to make suggestions about the type of polyethylene. To fully grasp these results, further investigation was performed in the next section.

#### 3.2.3. PCA Model

**Figure 3:**
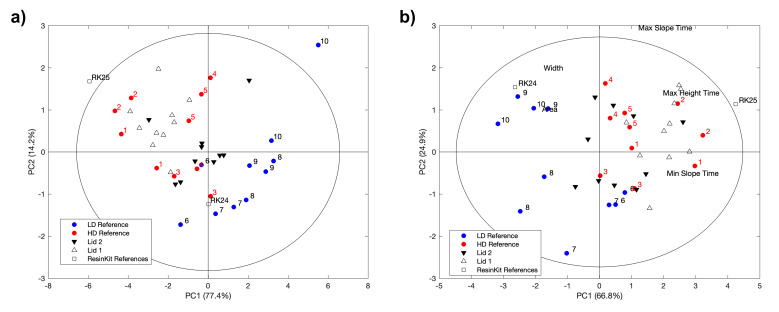
PCA for normalized EGA curves (Method A) (**a**) and curve parameters (Method B) (**b**). Two replicates for each irradiation time were considered, numbered for LDPE and HDPE references, unnumbered for lid 1 and lid 2. Projection of ResinKit^TM^ No. 24 and 25 is also reported.

To support the polymer type identification made with evolved gas analysis for lid 1 and clarify the nature for lid 2, the EGA thermograms were analyzed by PCA (Figure 3). Note that the PCA model was calibrated using the HDPE and LDPE samples during the several aging steps (numeration is 1(0 h)–2(110 h)–3(220 h)–4(550 h)–5(770 h) for HDPE, and 6(0 h)–7(110 h)–8(220 h)–9(550 h)–10(770 h) for LDPE). Lid 1 and lid 2 samples at the same aging intervals were then projected onto the model to observe the relative positions against the reference polymer samples. Initially considering Method A, the two first principal components accounting for more than 98% of the total EGA signal variance were examined regarding the ability for separating HDPE and LDPE reference samples. A scatter plot of the two components shows a separation between HDPE and LDPE, confirming that EGA patterns are indeed different for both polymer types, unaged and aged (Method A). In the score plot, the HDPE and LDPE samples formed two separate clusters, indicating not only differences between them but also that each type of polymer may yield slightly varied profiles, thus explaining the observed scatter of projections/data/results. The samples’ projection showed that lid 1 matched the HDPE sample, while lid 2 is placed in the middle of the graph with a closer match with HDPE than LDPE (Figure 3a). Considering the analysis using the extracted descriptors from EGA signals (Method B), LDPE is characterized by broader bands at lower temperatures (higher area and width, on the left of the graph), while HDPE bands are narrower, located at higher temperatures (max, min slope time, and time at maximum peak height are shorter, on the right) (Figure 3b). The same conclusions concerning the polymer nature of lid 1 and 2 can be drawn, as the sample projections of both lids over time are closer to the HDPE cluster. Although a low repeatability of the EGA method was observed (replicates for each irradiation time do not in general overlap in the score plot), the information extracted from the PCA method consolidates the interpretation by EGA for lid 1 and suggests that lid 2 is also made of HDPE. The Projection of ResinKit^TM^ samples No. 24 and 25 fits well with types of PE reference samples, ensuring the robustness of the PCA model.

### 3.3. β-Naphthol Synthetic Pigments: Reference Powders and Historical Plastic Samples

In this study, both EGA-MS and Py-GC/MS were used: (1) to characterize the thermal decomposition and the Py-markers of the reference pigment powders before and after aging, and (2) to investigate the presence of these markers in the historical samples, and eventually identify degradation products due to aging.

#### 3.3.1. EGA-MS Results

**Figure 4:**
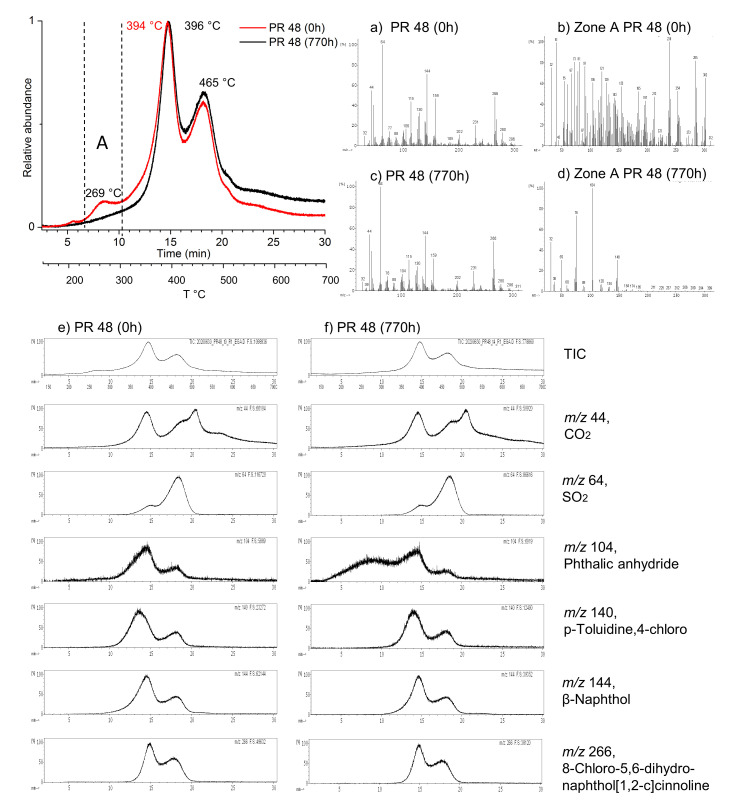
EGA-curves of the pigment PR 48:2 at 0 h (no aging) and at 770 h and related mass spectra: (**a**) average mass spectrum of PR 48:2 at 0 h, (**b**) average mass spectrum of zone A for PR 48:2 at 0 h, (**c**) average mass spectrum of PR:2 48 at 770 h, (**d**) average mass spectrum of zone A for PR 48:2 at 770 h, (**e**) total ion current (TIC) and extracted ions for PR 48:2 at 0 h, and (**f**) total ion current (TIC) and extracted ions for PR 48:2 at 770 h. The ion trends are scaled to the largest peak in each plot.

**Figure 5:**
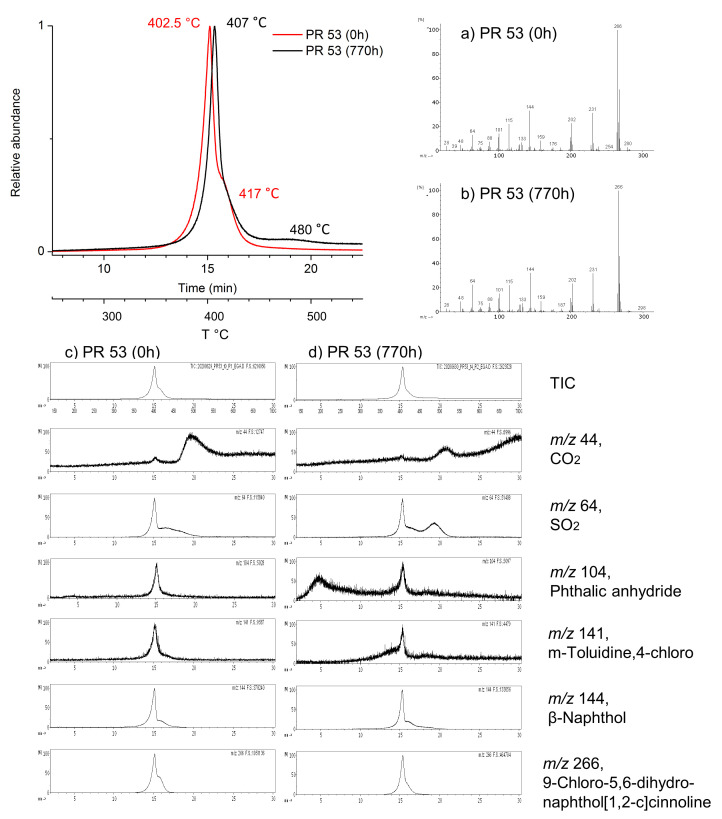
EGA-MS curves of the pigment PR 53:1 at 0 h (no aging) and 770 h and related mass spectra: (**a**) average mass spectrum of PR 53:1 at 0 h, (**b**) average mass spectrum of PR 53:1 at 770 h, (**c**) total ion current (TIC) and extracted ions for PR 53:1 at 0 h, (**d**) total ion current (TIC) and extracted ions for PR 53:1 at 770 h. The ion trends are scaled to the largest peak in each plot.

The EGA-MS analysis of the powder pigments at 0 h showed their different molecular structures, presenting different TIC thermograms and volatile products evolved in the temperature range 100–700 °C. In addition, the shift and appearance/disappearance of EGA peaks between the unaged and aged pigment powders indicate the occurrence of light-induced molecular changes (Figure 4 and Figure 5).

Before aging (0 h), PR 48:2 is characterized by three main peaks: the first at 269 °C, the second at 394 °C, and the third at 465 °C. After aging (770 h), the first peak disappeared, the second shifted to 396 °C, and the third remained at the same temperature. Considering the average mass spectra, before and after aging, the samples did not show significant differences (Figure 4a,c). However, focusing on the zone A between 240 and 300 °C, at 0 h, several compounds evolved in this temperature range (Figure 4b), while after 770 h, the majority of those were lost, but the typical ions of phthalic anhydride (C_8_H_4_O_3_: *m*/*z* 50, 76, 104, 148) [98] could be identified (Figure 4d).

From [77], the main Py-markers of PR 48:2 are *p*-toluidine,3-chloro, *β*-naphthol, and 8-chloro-5,6-dihydronaphthol[1,2-c]cinnoline. Moreover, it is also known that during the thermal degradation of sulfonated organic compounds, CO_2_ and SO_2_ are produced [99,100]. Therefore, to better understand the light-induced changes in the pigment composition, the specific ions of the aforementioned reference compounds, including phthalic anhydride, were extracted from the EGA-curves before (0 h) and after aging (770 h) (Figure 4e,f), in particular: *m*/*z* 44 for CO_2_, *m*/*z* 64 for SO_2_, *m*/*z* 104 for phthalic anhydride, *m*/*z* 140 for *p*-toluidine,3-chloro, *m*/*z* 144 for *β*-naphthol, and *m*/*z* 266 for 8-chloro-5,6-dihydronaphthol[1,2-c]cinnoline. The extracted thermograms for the CO_2_, SO_2_, and pigment-markers before and after aging showed similarities. The two major bands of the TIC trends were associated with the pigment Py-markers, while SO_2_ was mainly found at 465 °C. The TIC trends were also characterized by a constant CO_2_ emission prolonged until 700 °C. The most significant difference was observed in the extracted thermograms of phthalic anhydride. The appearance of a broad band between 160 and 320 °C after aging clearly indicates that this phthalic compound is a degradation product.

By contrast, PR 53:1 at 0 h was mainly characterized by one peak at 402.5 °C, and a shoulder around 417 °C. After aging (770 h), the peak moved to higher temperatures (407 °C), with the shoulder disappearing, and a small band appearing around 480 °C (Figure 5).

Since PR 53:1 and PR 48:2 share the same basic skeleton structure with exchange of the—chlorine and—methyl substituents on the benzene ring (Supplementary Table S2), the ion extraction of PR 53:1 considered similar compounds to those selected for the interpretation of PR 48:2, however *m*-toluidine,4-chloro (*m*/*z* 141) and 9-chloro-5,6-dihydronaphthol[1,2-c]cinnoline (*m*/*z* 266) [77] were included. Comparing the extracted thermograms of PR 53:1 before and after aging, subtle differences were observed (Figure 5). The thermal evolutions of *m*-toluidine,4-chloro and *β*-naphthol did not present substantial changes, in contrast to 9-chloro-5,6-dihydronaphthol[1,2-c]cinnoline, which showed the disappearance of the shoulder at 417 °C after 770 h of aging (Figure 5c,d). Also associated with aging, a small increase of CO_2_ emission at higher temperatures (550–700 °C) and a SO_2_ emission responsible for the new broad and weak band at 480 °C were detected. The emission of phthalic anhydride at around 200 °C, as also detected for PR 48:2 in the same temperature range after aging, suggests the formation of the same degradation product for both *β*-naphthol pigments.

No evidence of the red pigments was visible in the thermograms of the historical lids (Figure 2), although this method is suitable for their identification, as showed in Figure 4 and Figure 5 for organic pigments’ characterization. The very abundant contribution of the polymer to the thermogram could probably mask the pigment’s EGA peaks. Normally, in plastic coloring, pigments are used in very small concentrations (0.1–2.0% and up to 5% for special requests) depending on the plastic formulation and end use application [5].

#### 3.3.2. Py-GC/MS Results

The Py-GC/MS analysis on the reference pigment powders detected the same principal Py-markers [77] previously identified by EGA-MS, confirming the presence of phthalic anhydride **(1)** as a photodegradation product in both pigments (Table 1). Further photodegradation compounds were detected: phthalimide **(3)** in both pigments, and 1,3-indandione **(2)** only in PR 48:2. The complete list of the pyrolysis products of the red pigments is presented in Supplementary Table S4.

**Figure 6:**
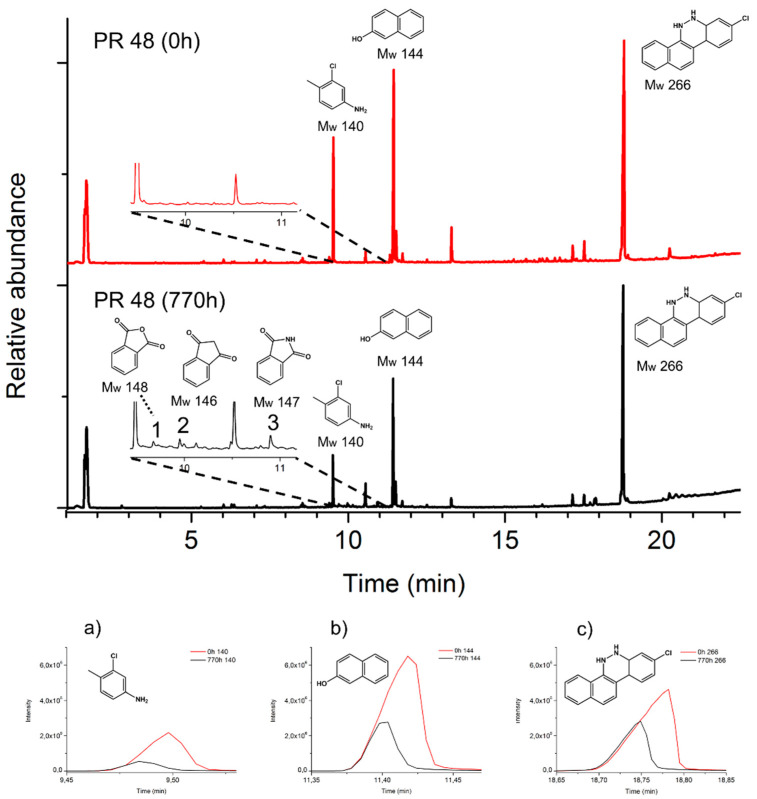
Top: normalized pyrograms of the red powder pigment PR 48:2. At 0 h, the main pyrolysis structures (M_w_ 140, M_w_ 144, and M_w_ 266) are observed; after aging (770 h), decay products (M_w_ 148, M_w_ 146, and M_w_ 147) are formed (Table 1). Bottom (**a**–**c**): principal extracted ions (base peaks) of the main pyrolysis products.

**Figure 7:**
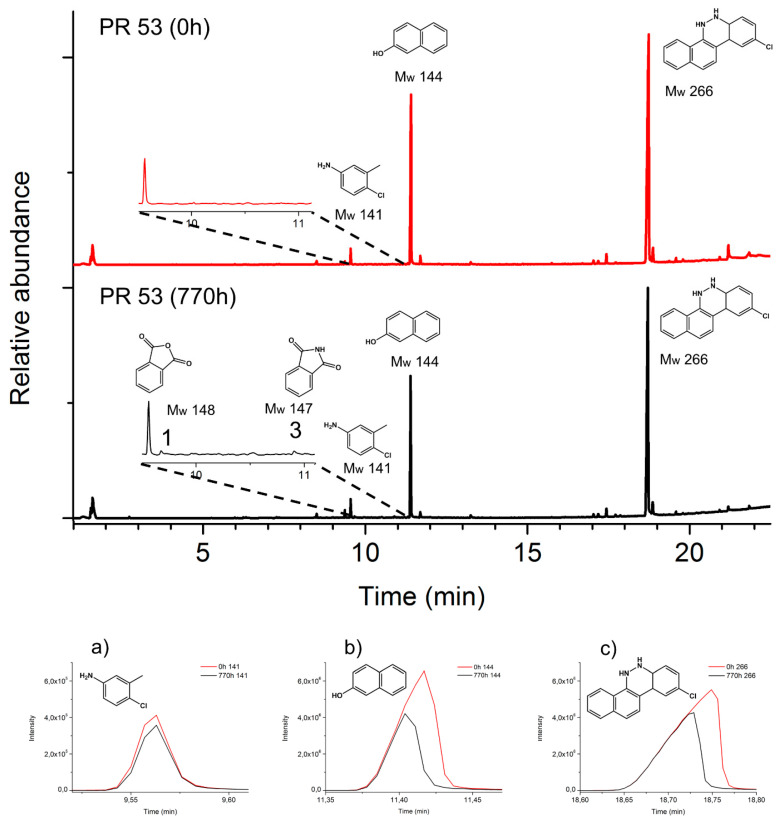
Top: normalized pyrograms of the red powder pigment PR 53:1. At 0 h, the main pyrolysis structures (M_w_ 141, M_w_ 144, and M_w_ 266) are observed; after aging (770 h), decay products (M_w_ 148 and M_w_ 147) are formed (Table 1). Bottom (**a**–**c**): principal extracted ions (base peaks) of the main pyrolysis products.

Figure 6 and Figure 7 show the pyrograms of the pigment powders before (0 h) and after aging (770 h). The main pyrolysis products of both pigments (Figure 6a–c and Figure 7a–c) decreased with irradiation.

Since no information related to the organic pigments could be extracted from EGA-MS analysis of the historical samples (possibly due to very low concentrations), their presence in the lids 1 and 2 was investigated by Py-GC/MS analysis.

**Figure 8:**
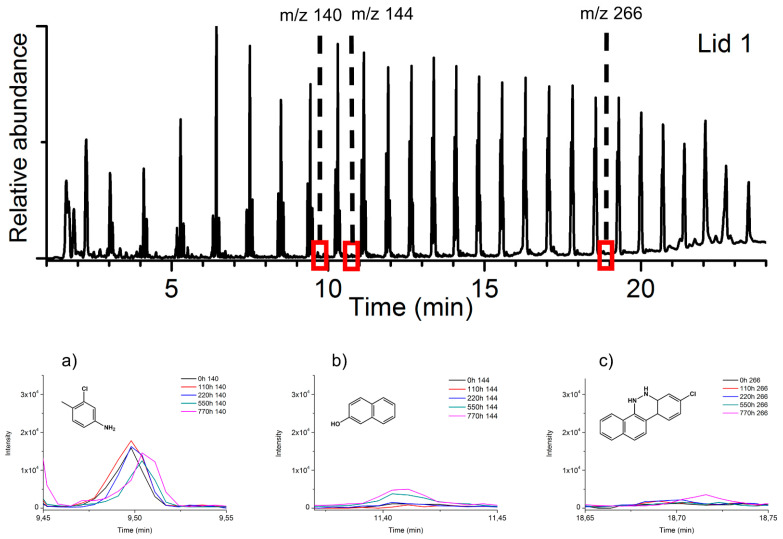
Normalized pyrogram of lid 1 at 0 h and related extracted ions (base peaks) of the main pyrolysis products of the pigment PR 48:2, M_w_ 140 (**a**), M_w_ 144 (**b**), and M_w_ 266 (**c**), during the several aging steps (0–110–220–550–770 h).

**Figure 9:**
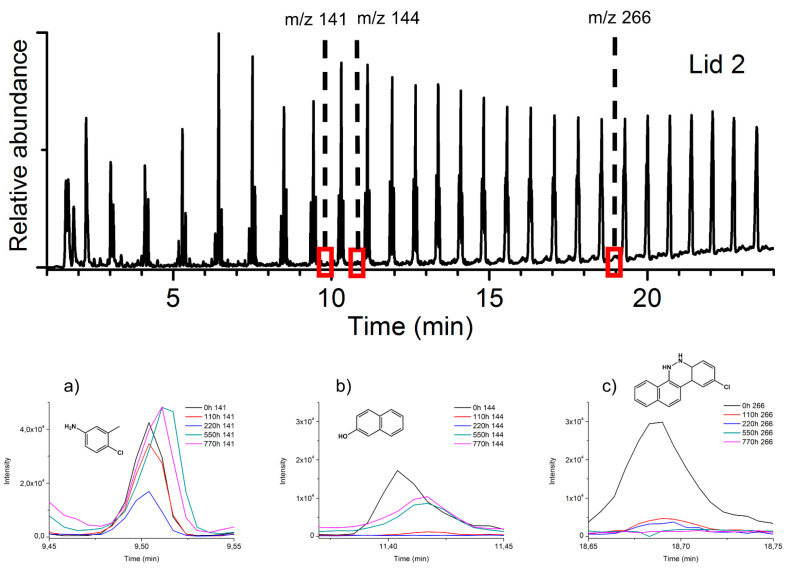
Normalized pyrogram of lid 2 at 0 h and related extracted ions (base peaks) of the main pyrolysis products of the pigment PR 53:1, M_w_ 141 (**a**), M_w_ 144 (**b**), and M_w_ 266 (**c**), during the several aging steps (0–110–220–550–770 h).

Considering the pyrolysis results obtained for the reference pigments, Figure 8 and Figure 9 show the pyrograms of the two lids, highlighting the extracted ions *m*/*z* 140, 144, 266, and *m*/*z* 141, 144, and 266 for PR 48:2 (Figure 8a–c) and PR 53:1 (Figure 9a–c), respectively. In lid 1 and lid 2, the signals of the three main Py-products from both pigments are relatively low when compared to those of the polymer. The ion *m*/*z* 140 deriving from PR 48:2 was easily detectable before and after aging (Figure 8a), while *m*/*z* 144 (Figure 8b) and 266 (Figure 8c) appeared in very small amount or even in traces. In lid 2, the three main Py-products were visible before aging, *m*/*z* 141 was still detectable afterwards (Figure 9a), whereas *m*/*z* 144 (Figure 9b) and 266 (Figure 9c) were less recognizable. No signals of the degradation products were recognized in the plastic lids.

### 3.4. Additives: Historical Samples

Considering the EGA-MS analysis, both historical samples showed no clear signal of any significant amounts of additives in the temperature range between 100 and 350 °C (Figure 2). Polymer additives are usually present at concentrations in the order of 0.1–1 % *w*/*w* [101] and, other than fillers, antioxidants, stabilizers, and UV light protectors are key and typically used for polyolefins.

The investigation of the presence of additives in the lids 1 and 2 was additionally performed by TD-GC/MS analysis. TD-chromatograms are depicted in Supplementary Figure S11 and the summary of the related volatile organic compounds is reported in Table 2. No additives were identified in the historical samples, confirming the EGA-MS results, while few compounds, mainly deriving from the PE chains (similar to unsaturated chains C18–C32 from Py analysis of PE) [76], were detected. As for the other additives, no signals concerning the presence of red pigments were found in the TD-chromatograms.

## 4. Discussion

### 4.1. Polymer Reference Matrix

HD- and LD-PE samples tended to yellow and crack as a consequence of the artificial aging. Both ATR-FTIR and EGA-MS measurements corroborated the occurrence of the polymer chain scission, which can be considered the main transformation of the macromolecular PE chain with aging. In detail, ATR-FTIR identified the vibrational markers of carboxylic acids, esters, lactones, vinyl, and *t*-vinylene functional groups. Additionally, the –CH_3_ end groups were observed for HDPE. The broadening and shift towards lower temperatures of the EGA-MS curves highlighted the creation of shorter polymer chains with photo-oxidation.

Both polyethylenes were susceptible to photo-oxidation, with LDPE to a higher extent, due to its molecular structure (branching). The identification of the PE type is thus considered key in providing reliable predictions of possible color change in PE plastics following exposure to light, as yellowing is more likely to occur in LDPE. In pristine condition, the distinction between HD and LD polyethylenes was proved possible by IR analysis. However, in aged samples, the identification of the polymer type is challenging because of the light-induced modification in the microstructure (i.e., chain scission and formation of carbonyl and vinyl groups). The application of the PCA method on the EGA signals enabled the extrapolation of useful data for the PE type identification. The two methods used to create the PCA model led to the identification of HDPE in both the historical lids.

### 4.2. β-Naphthol Pigment Powders

It has been proven that EGA-MS and Py-GC/MS can be effective techniques for the identification and characterization of the synthetic organic pigments PR 48:2 and PR 53:1, as well as their light-induced alterations.

In the EGA-MS measurements, the two *β*-naphthol pigments were characterized by different thermal zones of interest (both in number and position), which made them discernible. In addition, EGA-MS was able to detect differences in the pigment composition after aging (i.e., identification of phthalic anhydride as a degradation product, appearance/disappearance and shift of the signals). The application of EGA-MS analysis in the characterization of organic pigment is mostly unexplored in the cultural heritage field, and the EGA-MS profiles of pure *β*-naphthol pigment lakes were presented for the first time. As used for other heritage materials [102,103,104,105], EGA-MS in combination with multi-shot pyrolysis may represent a valid method for studying synthetic organic pigments. Besides the identification, their combination would allow the selective characterization of specific volatile fractions, such as additives and degradation products.

Between the MS techniques, Py-GC/MS is one of the most accepted analytical methods for the study of synthetic organic pigments, including hydrazone-azo pigments [77,106,107,108]. This technique does not require sample preparation and overcomes some practical limitations that organic pigments can present using liquid chromatography (LC) approaches, such as pigment extraction and the scarce solubility in organic solvents. In this study, the Py-GC/MS procedure enabled the identification of the pyrolysis products characteristic of the individual pigments, which were used as markers for the fading study in the historical plastics: *p*-toluidine,3-chloro (*m*/*z* 140) and 8-chloro-5,6-dihydronaphthol[1,2-c]cinnoline (*m*/*z* 266) for PR 48:2, *m*-toluidine,4-chloro (*m*/*z* 141) and 9-chloro-5,6-dihydronaphthol[1,2-c]cinnoline (*m*/*z* 266) for PR 53:1, and common *β*-naphthol (*m*/*z* 144) for both pigments.

Thanks to EGA-MS and Py-GC/MS analyses, phthalic derivatives, such as phthalic anhydride, 1,3-indandione, and phthalimide, were observed in small concentrations as degradation products after 770 h of artificial aging. These organic compounds were already detected as reaction intermediates in the photodegradation studies of the parent dye C.I. Acid Orange 7 [109,110,111]. However, these studies used conditions for the photoaging (presence of catalysts and/or λ_irr_ < 300 nm) far from conventional artificial aging performed in the cultural heritage field.

From a recent review [72], it is clear that little is known about the photodegradation mechanisms of *β*-naphthol pigments. The study of two parent dyes [72] allowed a preliminary insight into the factors affecting the *β*-naphthol fading. After irradiation of solutions under a polychromatic source (λ_irr_ ≥ 300 nm), phthalic acid and phthalates were detected as degradation products. Phthalic acid can be converted into its anhydride by a condensation reaction, and the two compounds were already detected in the light-induced degradation of certain dyes [110,112,113]. Information on the degradation mechanism of the *β*-naphthol pigments still needs clarification. Nevertheless, the fact that the photodegradation of both parent dyes and *β*-naphthol pigments includes the formation of phthalic derivatives and phthalates as decay products can inspire further mechanistic studies.

Based on the MS measurements, no significant concentrations of additives were detected in the reference pigment powders. This confirms that the degradation products identified after aging are mainly formed from the light-induced degradation of the organic pigment molecules, without the influence of other components (i.e., additives).

PR 53:1 seemed more sensitive to the light aging condition tested in this study than PR 48:2. This hypothesis is posited based on the dramatic color change (red to pale pink) of the neat PR 53:1 powder. Fading of PR 53:1 was also detected by Ghelardi et al. [70]. The identification of whitish phthalic derivatives [114] agrees with the brightening of the pigment powder color.

Darkening of PR 48 pigment-type lakes and other *β*-naphthol red lakes as a consequence of UV exposure was already observed [70,71]. The loss of the chromophore in favor of colorless degradation products may account for the fading of the pigment; however, its darkening needs further clarification. The formation of phthalic derivatives can be a valid indicator of photodegradation at the early stages.

### 4.3. Discoloration of the Historical Plastic Samples

The historical lids degraded severely during artificial aging, reaching an average value of the total color difference of ΔE*_ab_ ≈ 45 (Supplementary Table S3). Chemical and physical degradation resulted from a combination of several processes: yellowing of the PE polymer, fading of the red organic *β*-naphthol pigment, and embrittlement of the samples, which, in turn, led the material to collapse. Both yellowing and fading determined severe changes in the color appearance of the aged historical samples. The action of light in the presence of oxygen is *de facto* the fundamental degradation mechanism to which plastics are subjected during their lifetime [34,74]. Considering that the formulation of both lids is based on a polymer that yellows and pigments that fade with photo-oxidation, their photodegradation involved chemical changes of both polymer and organic pigments. Only the absorbed radiation can lead to chemical changes. This is a complex mechanism and the likely interplay between the polymer and organic pigment behavior still needs to be further explained. To rationalize the polymer photodegradation, the absorption of the organic pigments should be clarified with further research, as radiation λ ≥ 300 nm most probably would be absorbed by the lakes [72].

Compared to the aging of the reference polymers, similar conclusions can be generally made on the photodegradation of the PE historical matrices. This indicates that the degradation pathway and chromophores responsible for the PE yellowing would be comparable.

The aging experiments highlighted PR 48:2 and PR 53:1 as photosensitive pigments in PE. Fading of the historical lids can be related to the partial or total color loss of the pigment particles inside of the plastics, turning into a whitish or much lighter color (Supplementary Figures S3–S5). Fading of the PR 53:1-containing lid is in accordance with the brightening of the neat pigment powder. Although the solely PR 48:8 darkened, this was not observed in lid 1 (Supplementary Figures S3 and S4). This discrepancy between the neat powder and the plastic system may be associated with different mechanism(s) and/or rate(s) of degradation, which lead to the formation of colorless degradation products.

After only 770 h, a noticeable color change was visible for the pigment powders, while, dispersed in the plastic matrix, at 110 h, some particles had already lost the red color under the OM. This can indicate a greater degree of sensitivity to light by the pigment lakes in the plastic samples. The polymer matrix of both lids could either have increased the reaction rate or induced other degradation mechanisms that led to severe fading in a shorter time. This evidence will have to be confirmed in future research, wherein the light absorbed by the pigments should be quantified, or at least guarantee the similar absorbance values prior to irradiation, in order to provide a better rationalization of the chemical events.

Fading was also observed as a superficial phenomenon which, to a high extent, can induce the total color loss of the plastics. In the aged mock-ups studied by Stenger [71], the same observation was made.

The use of Py-GC/MS for the identification of organic pigments in plastics was already tested [39,40,115]. Py-GC/MS was recommended for the precise identification of the organic pigments in the plastic objects, although the discrimination of the pigment peak fragments in the complex pyrograms was challenging and it required an extensive spectral database. In this work, small component peaks originating from the pigment were detected between the large range and abundant peaks (mainly deriving from the pyrolysis of the polymer). The use of Py-GC/MS enabled the unequivocal identification of the red organic pigments in the historical lids.

To study the fading mechanisms, a relevant aspect to consider is the decrease of the Py-markers’ peaks. This demonstrated the consumption of the pigments in favor of the formation of the degradation products. Unfortunately, a clear decrease of the Py-markers’ peaks in the historical lids with aging was not observed, probably as a consequence of the sampling procedure. Each sample was taken on the surfaces exposed to irradiation under stereomicroscope. As observed with the OM (Supplementary Figure S5), the red pigment particles progressively lost their color with aging, making their sampling extremely difficult under the stereomicroscope. For the same reason and considering the relatively little Py-peaks of the organic pigments compared to PE signals (Figure 8 and Figure 9), degradation products such as phthalic anhydride and/or other intermediates were not recognized in the plastic lids. The relative abundance of the polymer with its several fractions, with high intensity, probably masked the degradation products present in small amounts, making the analysis of the MS signals unsatisfactory. To collect the discolored pigment particles in the PE matrix, the use of OM could have probably better supported the sampling procedure.

No organic and organometallic additives were detected in the historical lids, at least with a detectable concentration. Usually, few additives in relatively low concentration levels are required in the PE formulation, compared to other polymers [7,116,117]. Unfortunately, no historical documents on the formulation procedure are available from the Portuguese producer [118] to support our finding.

Nevertheless, it is important to mention that titanium dioxide, zinc sulfide, iron oxide-based pigments, and inorganic additives such as fillers (i.e., calcium carbonate, barium sulphate, silica) could have played a role in the plastic discoloration (Supplementary Table S1) [41]. Extensive literature is available concerning the photosensitizing and photoprotective influence of the two crystalline modifications of titanium dioxide (rutile and anatase) on the polymer degradation, including polyolefins [43,44,119,120,121,122], while little research was conducted on the photoactivity of the other inorganic compounds in polymeric systems [43,44,121].

Considering the private collection from where the historical lids were gathered, PR 53 was not found discolored in all the plastic objects [41]. Preliminary investigation on historical lid 3 (Supplementary Figure S12) by EGA-MS and TD-GC/MS identified a polystyrene (PS) matrix (Supplementary Figure S13) and some additives (Supplementary Figure S14). In detail, the light stabilizer drometrizol (peak n° 8), and the lubricants palmitic acid butyl ester and stearic acid butyl ester (peaks n° 9 and 10), were detected (Supplementary Table S5).

Further studies on the combined effect of polymer-additives on organic pigment degradation (and consequent plastic fading) are needed, and the methodology followed in this research is further suggested: (1) obtain the single reference materials: polymer, pigment(s), additive(s), and their mixed formulation in a plastic, (2) artificially age both sets of samples, and (3) characterize their color and molecular changes. For preparing adequate plastic samples, both historical and tailored formulations can be considered.

## 5. Conclusions

In this work, the discoloration of historical polyethylene (PE) samples colored with PR 48:2 and PR 53:1, with special emphasis on the fading of the *β*-naphthol pigment lakes, was studied for the first time. The study considered the individual susceptibility of (i) polymers and (ii) pigments to photooxidation, and their combined effect on a historical plastic formulation.

Light induced visible alterations in the reference polymers, reference pigments, and historical plastic samples. Particularly, the absorption of light caused long-term, cumulative, and irreversible chemical and physical changes in the historical plastics samples, visible through color variation and embrittlement, respectively. The artificial aging helped in simulating the mechanism(s) that induced severe fading—the ultimate effect of light exposure in the natural aging of pigmented plastics.

*β*-naphthol PR 48:2 and PR 53:1 pigment lakes proved to be light-sensitive in the historical PE objects. Isolated neat pigment powders showed a lower level of degradation than in the plastic environment. This leads to the interpretation that the plastic matrix (functioning as a binder) could have promoted the degradation of the pigments, even if their higher sensitivity due to natural aging cannot be excluded. The photo-oxidation and consequent fading of both organic pigments pose threats to museum and plastic heritage collections. As for other cultural heritage materials [123], light presents a duality in its interaction with plastics: it is essential for the perception and appreciation of the artifacts, but it, too, contributes to their degradation and damage.

Considering our understanding of degradation mechanisms of this polymeric system [73,74], namely the fact that although PE does not directly absorb the UV-Vis radiation that reaches earth, it can overcome homolytic scission due to the existence of hydroperoxide groups that are formed during its synthesis, processing, etc. [74], one can infer that the hydroperoxide groups are the chromophores responsible for the formation of carbonyl-based functions and double bonds resulting from main- and side-chain scission. During these degradation processes, which can lead to scission and crosslinking, powerful oxidizing groups such as OH· are also formed. Thus, we can predict that these types of radical groups have a profound impact on the degradation of organic colorants present in the polymer matrix, as exemplified in the degradation of anthraquinone- and indigo-based systems [123,124].

The implemented analytical strategy used to investigate the individual sensitivity of (i) polymers (i.e., yellowing) and (ii) pigments to light-induced discoloration (i.e., fading of PR53:1 and darkening of PR 48:2), allowed to conclude that their combined influence in the historical plastic formulation resulted in the fading of both organic pigments to a severe degree, dramatically affecting the appearance of the historical plastic samples. This strategy is therefore recommended for further heritage science investigations on the discoloration of historical plastics.

The combined effect of the plastic formulation was studied on historical samples. The fact that they were naturally aged might have influenced the results obtained, however, they provided the match with real plastic artifacts. To overcome this impasse, the results should be verified, for example, on well-preserved historical samples or recently made tailored formulations for investigating the chemical reactivity of both naphthol reds in PE matrix.

The analytical methods adopted in undertaking this research were found suitable for the study of plastic fading. Microscopy observations and MS analysis enabled the new insight into plastic discoloration and *β*-naphthols degradation, providing useful information on the changes at the molecular level of both reference samples and historical lids. In detail, EGA-MS clearly characterized the photoaging of the polymer, and Py-GC/MS was the most valid method in studying the organic pigment, both in powder and in historical plastic matrixes. The unequivocal identification of phthalic derivatives as degradation products by Py-GC/MS opens new routes for studying the photodegradation mechanism(s) of both organic pigments. The application of TD-GC/MS in analyzing plastic heritage has been explored only recently. Here, it was effective in identifying the plastic additives of lid 3 and excluding their presence in lid 1 and lid 2.

The characterization of degradation products by means of Py-GC/MS originating from the artificially aged organic pigments in the historical lids was not conclusive. Future work includes the improvement of the sampling procedure, the preparation of tailored formulations reproducing historical production methods, and the application of a more specific analysis, such as the compound’s selective heart-cut/pyrolysis-gas chromatography/mass spectrometry (HC/Py-GC/MS), to be performed with higher amounts of sample (e.g., 500 µg). This will support a deeper understanding of the fading mechanism through the application of Py-GC/MS.

## Figures and Tables

**Table 1:** Main pyrolysis products of the red azo-pigments PR 48:2 and PR 53:1 [77]. The degradation products are numbered and marked in *italic*.

Type	Pigment	*t*_R_/min	Pyrolysis Products	Main *m*/*z*
BONA pigment lakes	PR 48:2	9.5	*p*-Toluidine,3-chloro	77, 106, 140
		11.4	*β*-Naphthol	115, 144
		18.8	8-Chloro-5,6-dihydro-naphthol[1,2-c]cinnoline	202, 231, 266
		9.6	**(1)** *Phthalic anhydride*	*76*, *104*, *148*
		9.9	**(2)** *1,3-indandione*	*76*, *104*, *146*
		10.9	**(3)** *Phthalimide*	*76*, *104*, *147*
*β*-Naphthol pigment	PR 53:1	9.6	*m*-Toluidine,4-chloro	77, 106, 141
lakes		11.4	*β*-Naphthol	115, 144
		18.7	9-Chloro-5,6-dihydro-naphthol[1,2-c]cinnoline	202, 231, 266
		9.6	**(1)** *Phthalic anhydride*	*76*, *104*, *148*
		10.9	**(3)** *Phthalimide*	*76*, *104*, *147*

**Table 2:** Volatile organic compounds detected in the red lids 1 and 2 (characteristic ions in mass spectra: molecular weight, M_w_, in bold and base peak underlined).

Peak Number	Compound	*m*/*z*
1	1-Octadecene (C_18_H_36_)	41, 55, 69, 83, 97, 111, 125, **252**
2	Similar to n-eicosene (C_20_H_40_)	43, 55, 69, 83, 97, 111, 125, **280**
3	Similar to 1-docosene (C_22_H_44_)	43, 57, 69, 83, 97, 111, 125, **308**
4	Similar to 1-tetracosene (C_24_H_48_)	43, 57, 69, 83, 97,111, 125, **336**
5	Similar to 1-hexacosene (C_26_H_52_)	43, 57, 69, 83, 97, 111, 125, **364**
6	Similar to 1-octacosene (C_28_H_56_)	43, 57, 69, 83, 97, 111, 125, **392**
7	Similar to 1-triacontene (C_30_H_60_)	43, 57, 69, 83, 97, 111, 125, **420**
8	Similar to 1-dotriacontene (C_32_H_64_)	43, 57, 69, 83, 97, 111, 125, **448**

## Supplementary Materials

The following are available online at https://www.mdpi.com/article/10.3390/polym13142278/s1, Figure S1: Arrangement of samples during the aging experiment: (a) historical and polymer reference samples, (b) pigment powders, (c) all samples inside of the chamber. Figure S2: L*, a*, b* values in 3-dimensional CIELab76 Color Space during aging: (a) HDPE, (b) LDPE, (c) lid 1, (d) lid 2. Projections of the points along the L* vertical axis and a* and b* perpendicular horizontal axes are also reported. The arrows define the color change over time. Figure S3: Stereomicroscope images of the plastic samples during the aging (25x). The pictures show the exposed areas. Figure S4: Stereomicroscope images taken from the side of the lids 1 and 2 at 0 and 550 h (ca.1 mm thickness). The exposed areas correspond to the upper part of the sample. Figure S5: Microscopy images of the red particles/aggregates dispersed in the polymeric matrix under reflected visible light (dark field) of the lid 2 sample. Images were collected directly from the sample in situ. Figure S6: Stereomicroscope images of the pigment powders at 0 and 770 h. Figure S7: ATR-FTIR spectra of lid 1 and lid 2 samples (top, blue lines). Vibrational spectra of the polymer references HDPE and LDPE are reported for comparison (bottom, black lines). Figure S8: ATR-FTIR spectra of HDPE, LDPE, lid 1, and lid 2 samples at t0 (detail of the interval 1300–1430 cm^−1^). Figure S9: ATR-FTIR spectra of HDPE (a), LDPE (b), lid 1 (c), and lid 2 (d) samples over aging. The arrows’ direction indicates the increase or decrease of IR bands’ intensity with increasing aging. Figure S10: ATR-FTIR spectra of the (a) HDPE and (b) LDPE with magnification of the 1300–1430 cm^−1^ range over aging. Figure S11: TD-chromatograms of lid 1 (up) and lid 2 (bottom). See Table 2 for peak identification. Figure S12: Historical sample presenting PR 53 not discolored. PS lid 3 on the bottom. Figure S13: Top: normalized EGA-MS curves of the red PS lid 3 and bottom: related average mass spectrum. Figure S14: TD-chromatogram of the red PS lid 3. For the peak identification, see Table S5. Table S1: Summary of multi-analytical analysis of polymer, red pigments, and white pigments/fillers from [1]. Table S2: Molecular structures and acronyms of the synthetic organic pigments studied. Table S3: Colorimetric coordinates of HDPE, LDPE, lid 1, and lid 2 at each irradiation time. Total color difference Δ*E**_ab_ (CIE1976), ΔE_00_ (CIEDE2000) between 0 and 770 h are reported. Table S4: List of pyrolysis products from the pigment powders PR 48:2 and PR 52:1 before (0 h) and after aging (770 h). Characteristic ions in mass spectra: molecular weight (M_w_) bold, base peak underlined. Table S5: Main volatile organic compounds detected in the red PS lid 3 (characteristic ions in mass spectra: molecular weight, M_w_, in bold and base peak underlined). Additives are marked in *italic*.
