# Supplementary material for: Discoloration of Historical Plastic Objects: New Insight into the Degradation of β-Naphthol Pigment Lakes
Source: Polymers (Basel). 2021 Jul 12;13(14):2278. doi: 10.3390/polym13142278 (PMC8309268; doi:10.3390/polym13142278)
Supplement: Supplementary file 1 [file polymers-13-02278-s001.zip › polymers-1197389-supplementary.pdf]

Supporting Information

Discoloration of Historical Plastic Objects: New Insight into the Degradation of *β*-Naphthol Pigment Lakes

Anna Micheluz [1], Eva Mariasole Angelin [2,*], João Almeida Lopes [3], Maria João Melo [2] and Marisa Pamplona [1,*]

1 Conservation Science Department, Deutsches Museum, Museumsinsel 1, 80538 Munich, Germany; a.micheluz@deutsches-museum.de (A.M.)
2 Department of Conservation and Restoration and LAQV-REQUIMTE, NOVA School of Science and Technology, NOVA University Lisbon, 2829-516 Caparica, Portugal; mjm@fct.unl.pt (M.J.M)
3 iMed.ULisboa-Research Institute for Medicines, Faculty of Pharmacy, University of Lisbon, Av. Prof. Gama Pinto, 1649-003 Lisbon, Portugal; jlopes@ff.ulisboa.pt (J.A.L.)
* Correspondence: e.angelin@campus.fct.unl.pt (E.M.A.); m.pamplona@deutsches-museum.de (M.P.)

Contents

Colorimetric measures……………………………………………………………………………2
Figures…..……...…………………………………………………………………………………2
Tables……………………………………………………………………………………………11
References…………………………………………………………………………………...…14

Colorimetric measures

During color measurement significant differences with the 0h are indicated for both PE reference samples and historical lids. In detail, stepwise increase in the positive b* values of HDPE and LDPE samples was observed indicative of yellowing (**Figure S2a,b**). The historical lids share the same tendency with their positive a* and b* coordinates decreasing towards more achromatic values as L* increases (**Figure S2c,d**). This trend is easily explained because the red pigments of the historical samples faded. L*a*b* coordinates and total color variation between 0h and 770h of irradiation are reported in **Table S3**. The most dramatic color change was observed for lid 1 and 2 (ΔE*ab > 45). Lid 2 was partially discolored before irradiation, and its color faded completely with the photoaging (**Figure S3**). Interestingly, it presents fading until 220h while after the only polymer contributes to color change with yellowing symptoms **(Figure S2d)**.

Figures

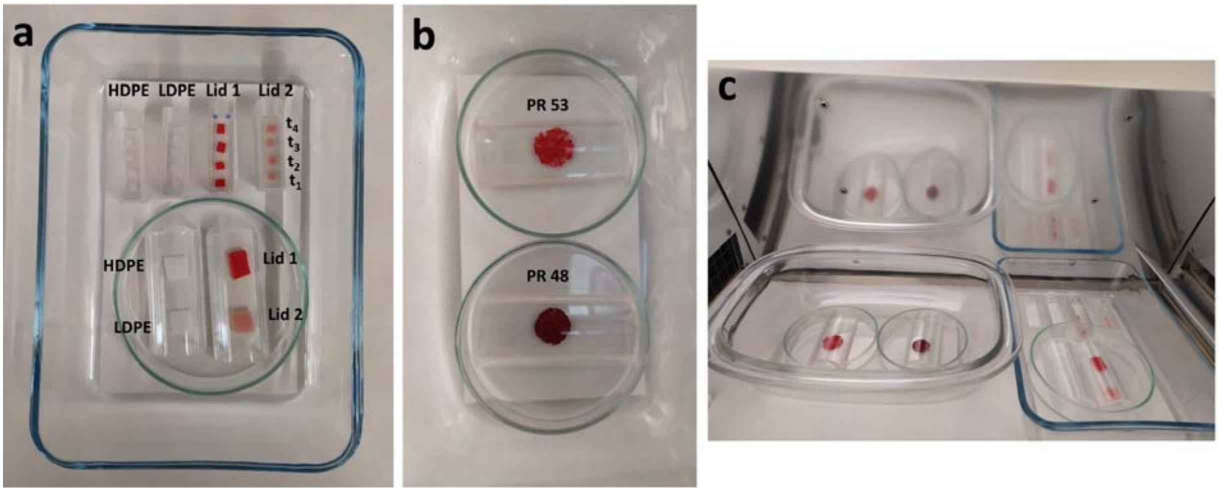

Figure S1. Arrangement of samples during the aging experiment. a) historical and polymer reference samples, b) pigment powders, c) all samples inside of the chamber.

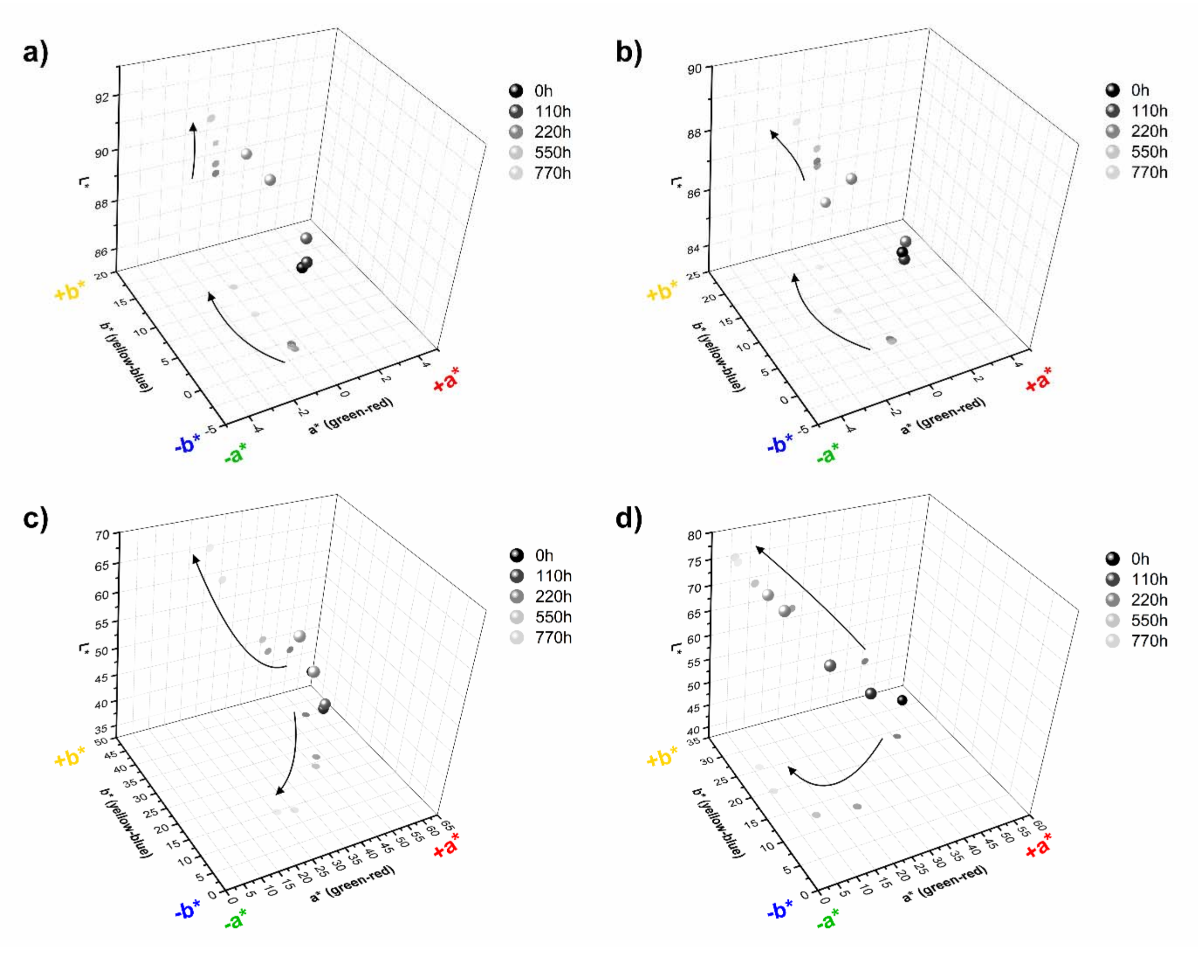

Figure S2. L*, a*, b* values in 3-dimensional CIELab76 Color Space during aging: (a) HDPE, (b) LDPE, (c) lid 1, (d) lid 2. Projections of the points along the L* vertical axis and a* and b* perpendicular horizontal axes are also reported.The arrows define the color change over time.

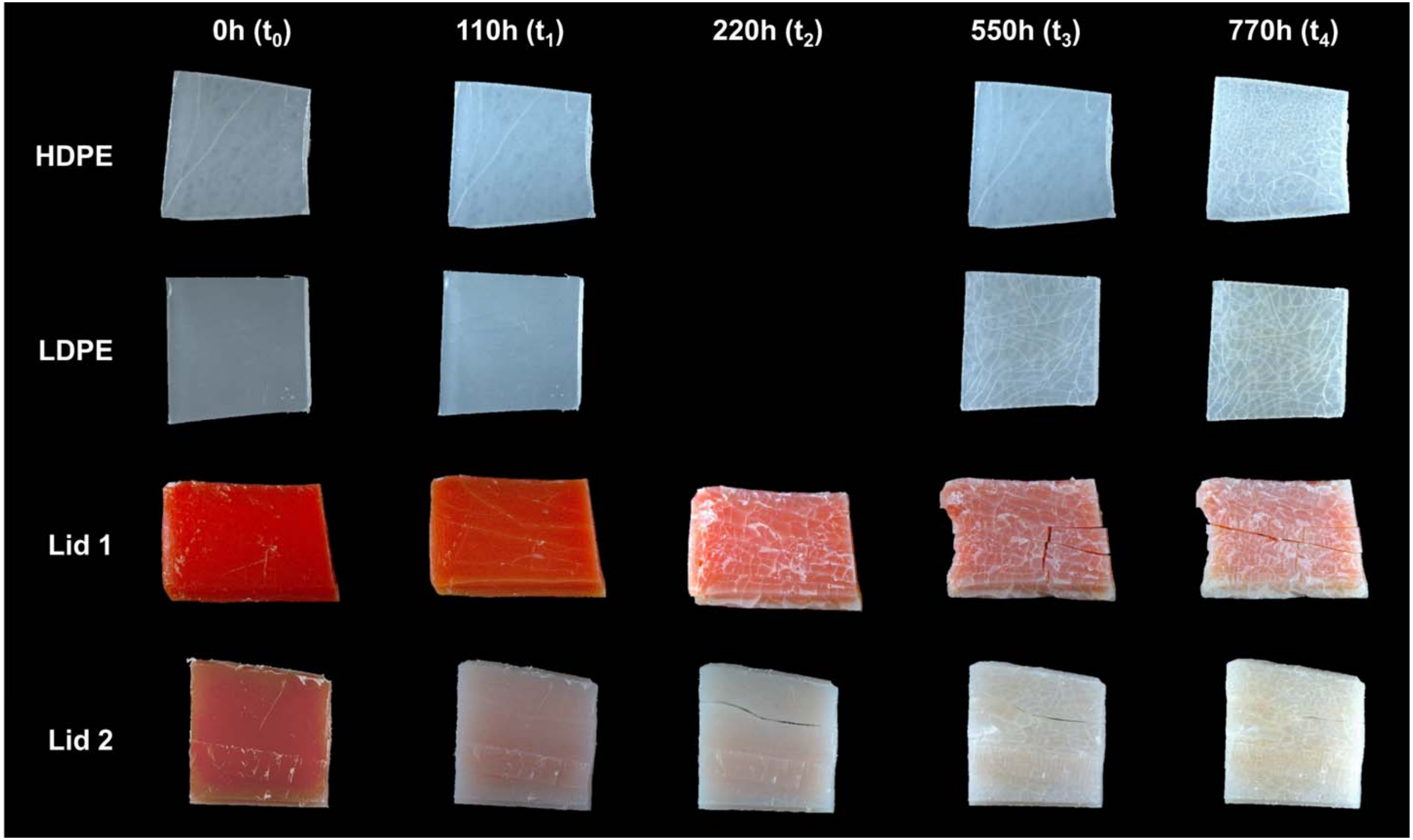

Figure S3. Stereomicroscope images of the plastic samples during the aging (25x). The pictures show the exposed areas.

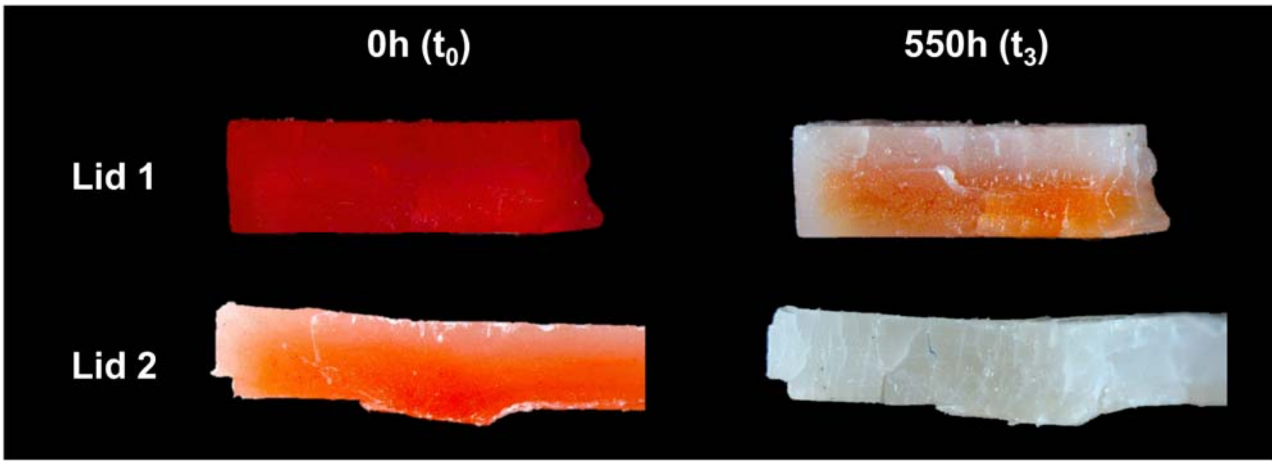

Figure S4. Stereomicroscope images taken from the side of the lids 1 and 2 at 0h and 550h (ca.1 mm thickness). The exposed areas correspond to the upper part of the sample.

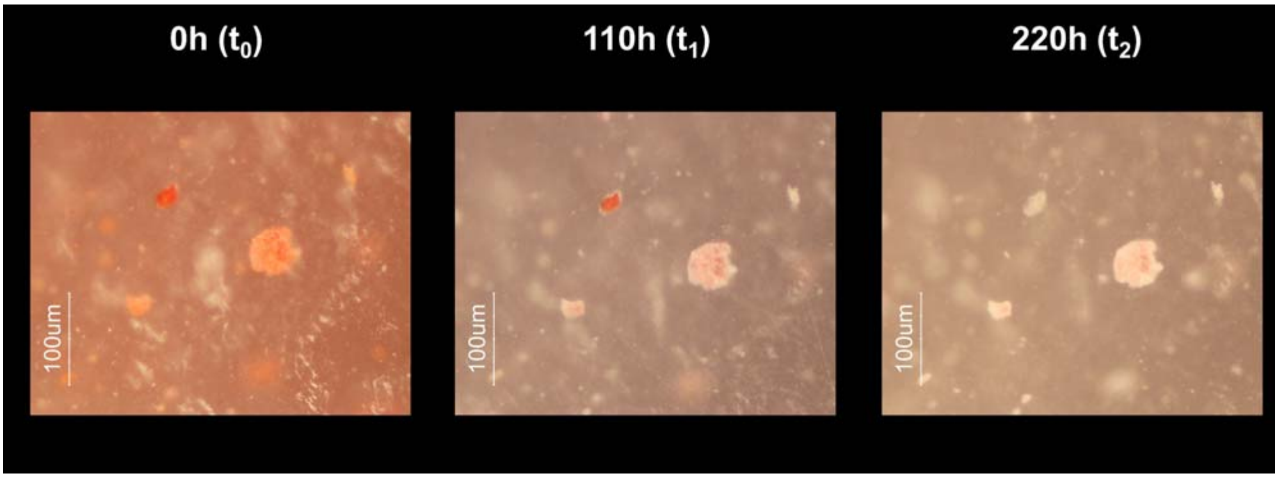

Figure S5. Microscopy images of the red particles/aggregates dispersed in the polymeric matrix under reflected visible light (dark field) of lid 2 sample. Images were collected directly from the sample in situ.

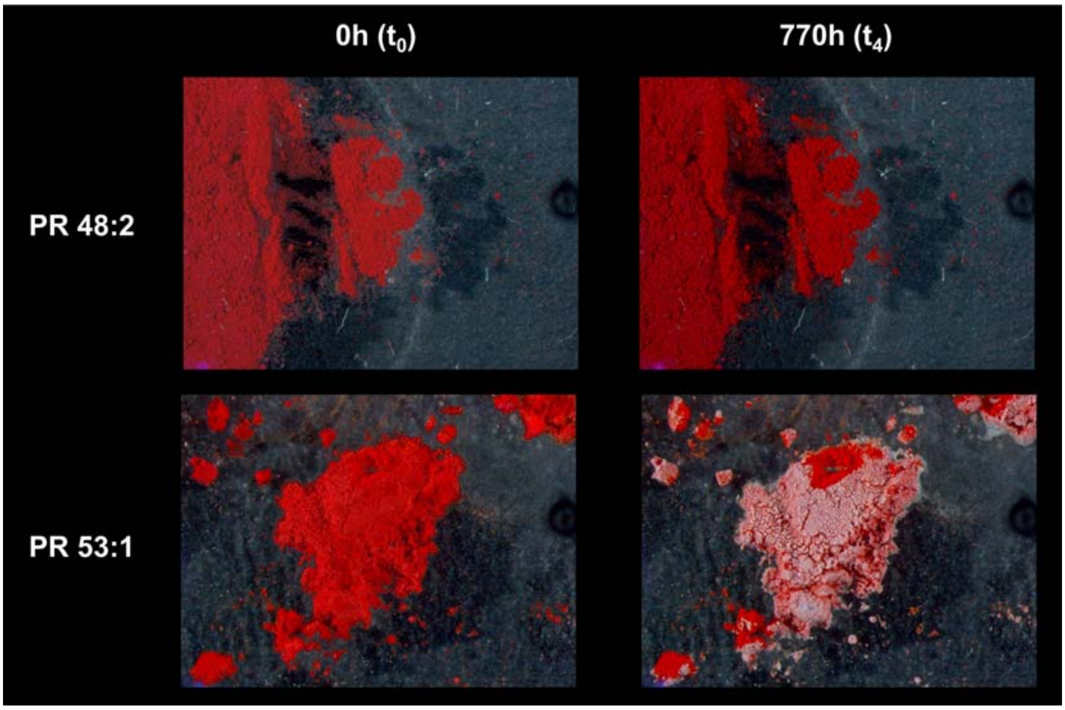

Figure S6. Stereomicroscope images of the pigment powders at 0h and 770h.

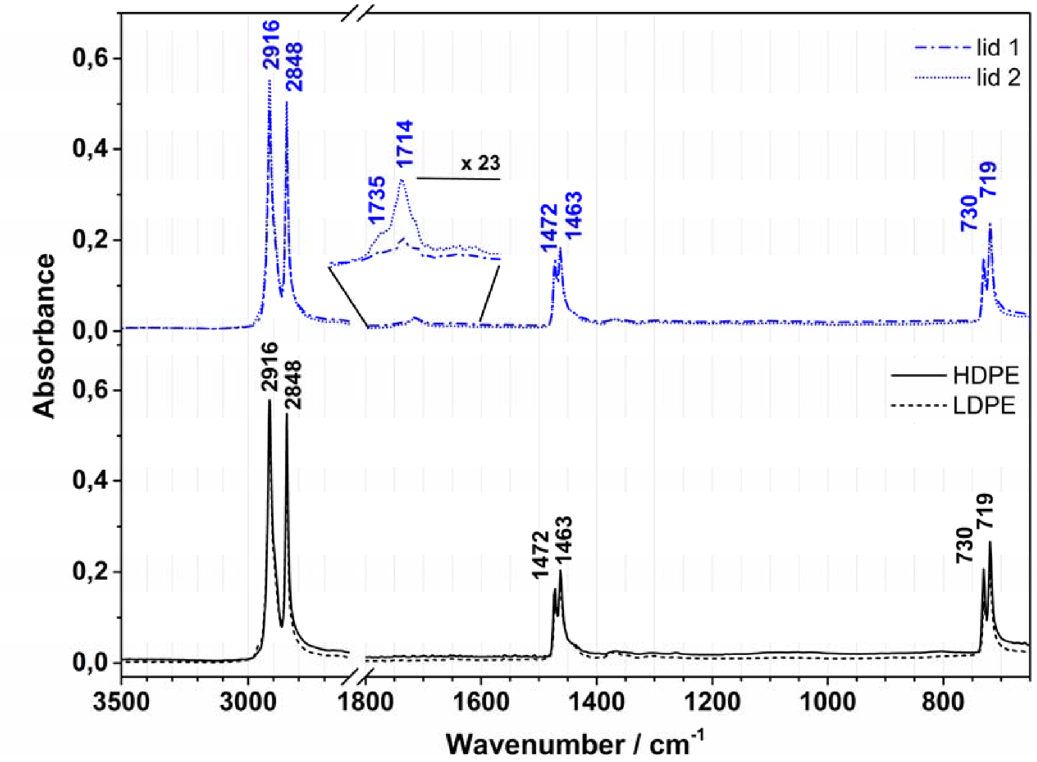

Figure S7. ATR-FTIR spectra of lid 1 and lid 2 samples (top, blue lines). Vibrational spectra of the polymer references HDPE and LDPE are reported for comparison (bottom, black lines).

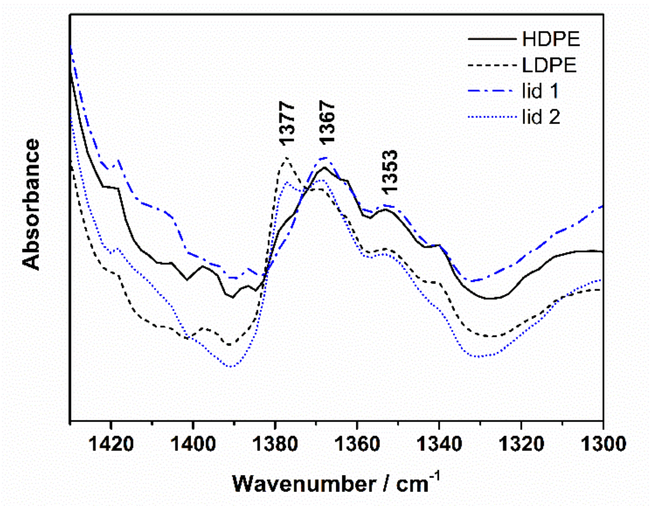

Figure S8. ATR-FTIR spectra of HDPE, LDPE, lid 1 and lid 2 samples at t_0 (detail of the interval 1300–1430 cm^{-1}).

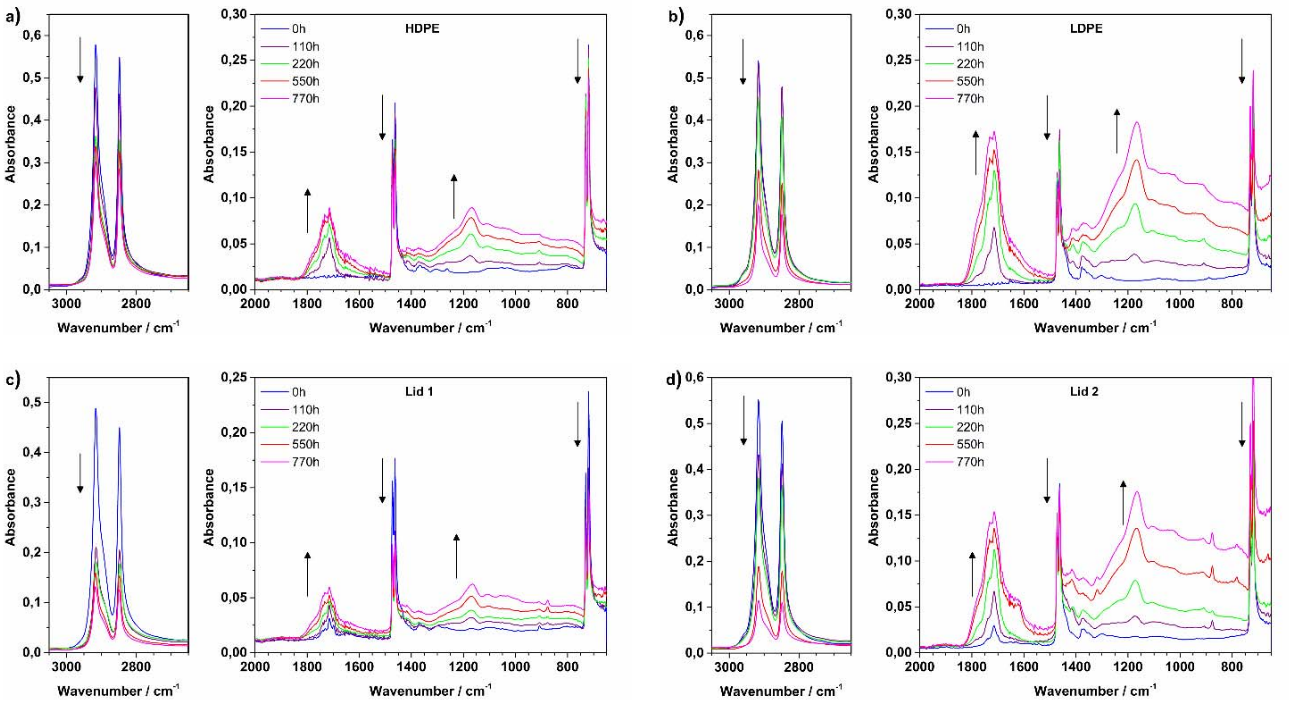

Figure S9. ATR-FTIR spectra of HDPE (a), LDPE (b), lid 1 (c) and lid 2 (d) samples over aging. The arrows' direction indicates the increase or decrease of IR bands intensity with increasing aging.

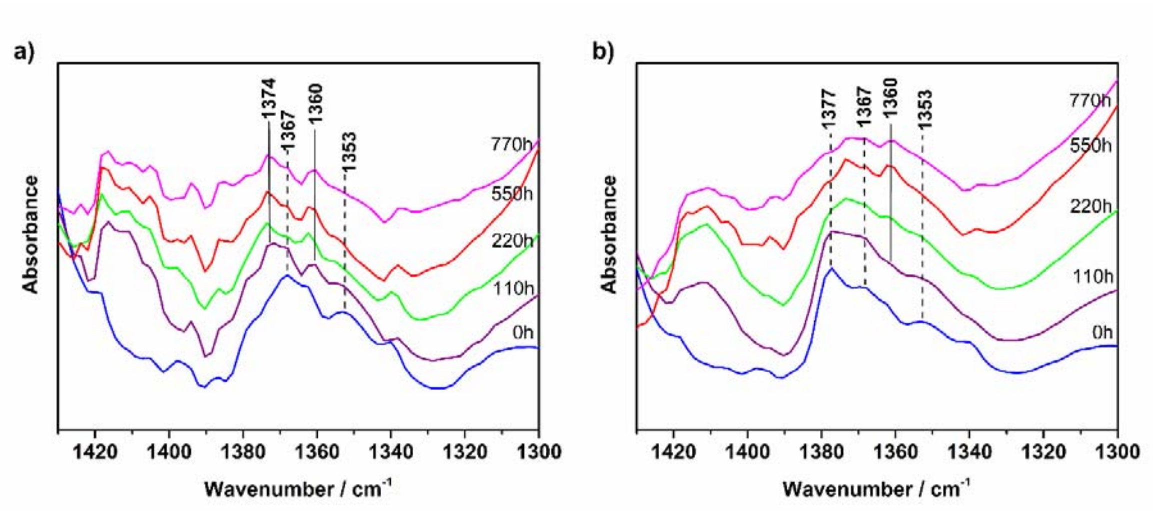

Figure S10. ATR-FTIR spectra of the (a) HDPE and (b) LDPE with magnification of the 1300–1430 cm^{-1} range over aging.

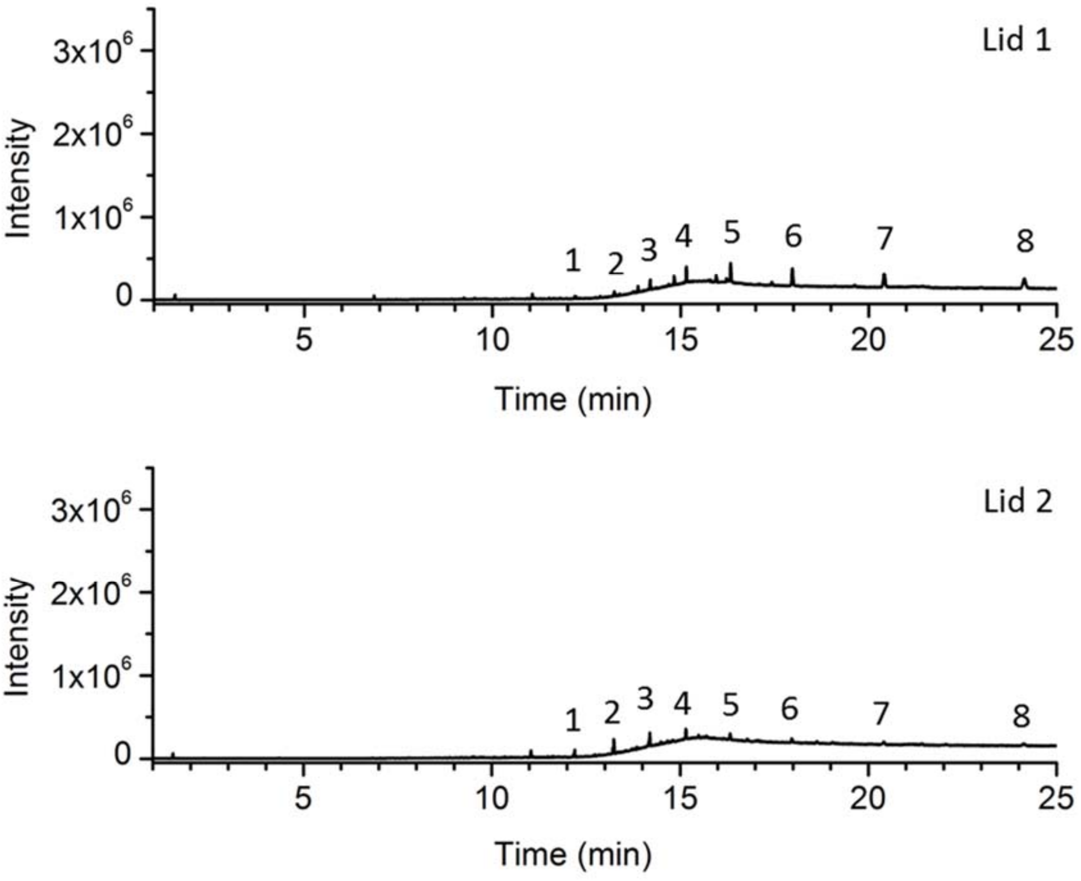

Figure S11. TD-chromatograms of lid 1 (up) and lid 2 (bottom). See **Table 2** for peak identification.

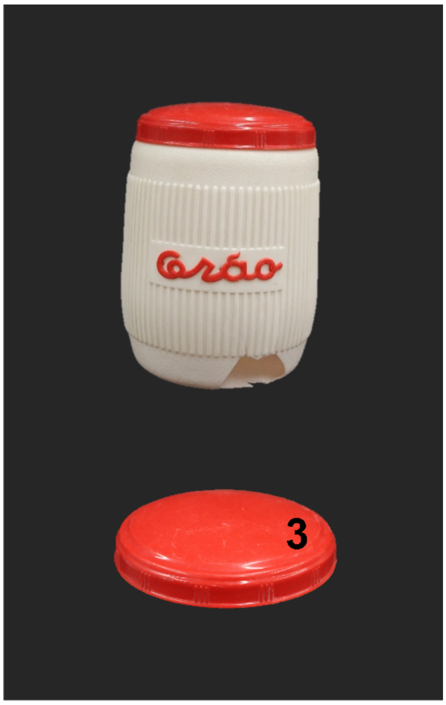

Figure S12. Historical sample presenting PR 53 not discolored. PS lid 3 on the bottom.

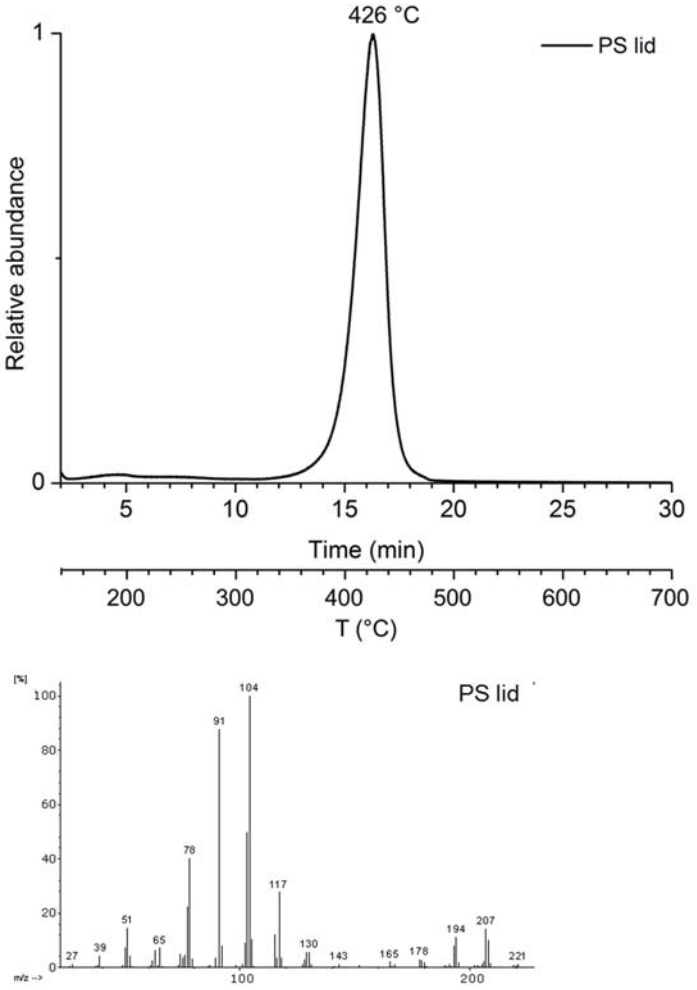

Figure S13. Top: normalized EGA-MS curves of the red PS lid 3 and bottom: related average mass spectrum.

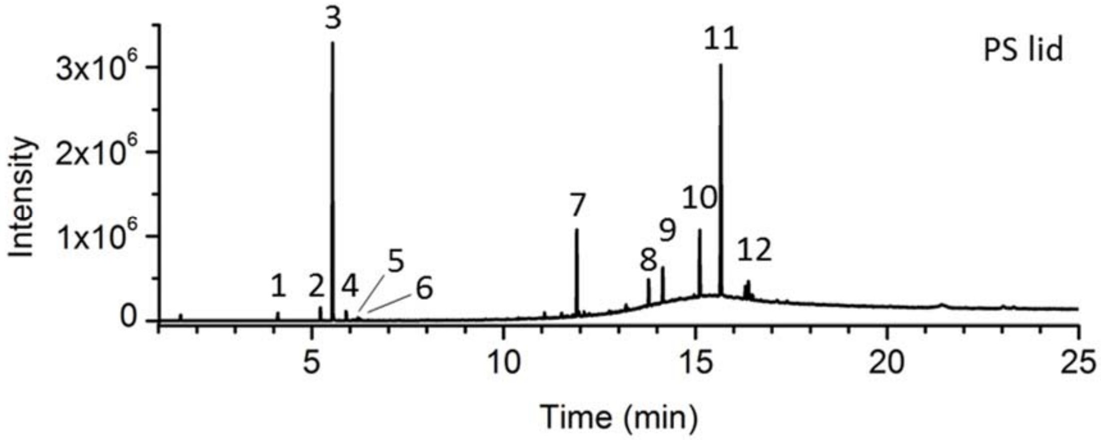

Figure S14. TD-chromatogram of the red PS lid 3. For the peaks identification, see **Table S5**.

Tables

Table S1. Summary of multi-analytical analysis of polymer, red pigments and white pigments / fillers from [1].

Historical sample	Plastic polymer	Elemental composition	Red organic pigments	Red inorganic pigments	White pigments / fillers
Lid 1	PE	Ti, Ca, Zn, Fe, K, Cl, Si	PR 48:2	PR 101	-
Lid 2	PE	Ti, Ca, Zn, Fe, Ba	PR 53:1	-	PW (R)

Note. PR: Pigment Red, PW: Pigment White, R: rutile crystalline form

Table S2. Molecular structures and acronyms of the synthetic organic pigments studied.

C.I. Generic name	Common name	C.I. Constitution number	Class[a]	Structure[b]
PR 48:2	Calcium Red 2B	15865:2	BONA pigment lake	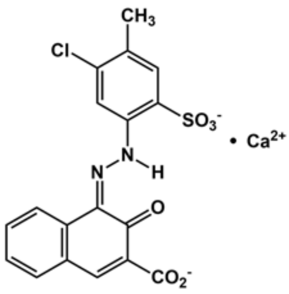
PR 53:1	Lake Red C	15585:1	*β*-naphthol pigment lake	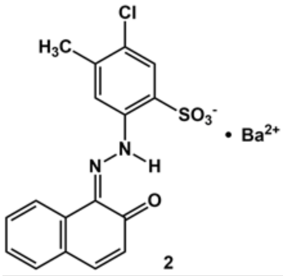

Note. PR: Pigment Red, [a] Classification of organic pigments based on the chemical structure as presented in [2]. [b] Colorants based on 1-arylhydrazone-2-naphthol skeleton, such as *β*-naphthol pigments, undergo a tautomeric rearrangement between hydrazone/keto and azo/enol forms. Molecular structures of the organic red pigment are depicted in the hydrazone/keto form which is accepted as the major tautomer [2].

Table S3. Colorimetric coordinates of HDPE, LDPE, lid 1 and lid 2 at each irradiation time. Total color difference ΔE^*_{ab} (CIE1976), ΔE_{00} (CIEDE2000) between 0h and 770h are reported.

	HDPE			LDPE			Lid 1			Lid 2		
	L*	a*	b*	L*	a*	b*	L*	a*	b*	L*	a*	b*
0h (t_0)	88.17	-0.41	2.04	86.14	-0.01	3.92	41.55	55.61	40.56	46.02	45.99	23.65
±	0.12	0.01	0.02	0.05	0.01	0.02	0.04	0.06	0.07	0.05	0.20	0.08
110h (t_1)	88.57	-0.44	1.28	85.96	0.03	3.53	42.37	47.87	26.59	61.87	22.12	12.04
±	0.06	0.02	0.01	0.01	0.01	0.01	0.03	0.15	0.23	0.05	0.01	0.06
220h (t_2)	89.42	-0.51	1.67	86.60	-0.06	3.40	45.08	45.54	23.89	68.67	11.51	12.92
±	0.51	0.01	0.03	0.07	0.01	0.01	0.02	0.05	0.06	0.29	0.10	0.06
550h (t_3)	90.43	-0.85	7.65	87.69	-1.14	11.20	58.45	30.51	13.74	74.59	5.97	19.97
±	0.80	0.01	0.04	0.25	0.02	0.04	0.09	0.08	0.16	0.24	0.01	0.32
770h (t_4)	90.45	-0.81	12.54	85.44	-0.41	20.07	64.62	25.87	14.74	73.47	6.81	25.67
±	0.08	0.04	0.15	0.16	0.01	0.04	0.06	0.05	0.14	0.28	0.04	0.97
$\boldsymbol{\Delta E^*_{ab}}$			**10.75**			**16.17**			**45.64**			**47.88**
$\boldsymbol{\Delta E_{00}}$			**8.04**			**10.50**			**25.74**			**33.94**

Table S4. List of pyrolysis products from the pigment powders PR 48:2 and PR 52:1 before (0h) and after aging (770h). Characteristic ions in mass spectra: molecular weight (M_w) bold, base peak underlined.

t_R / min	Compound	*m/z*	PR48 (0h)	PR48 (770h)	PR53 (0h)	PR53 (770h)
1.56	CO_2	28, **44**	x	x	x	x
1.60	Sulfur dioxide	32, 48, **64**	x	x	x	x
2.73	Benzene	39, 51, **78**	x	x	x	x
5.27	Styrene	51, 78, **104**	x	x	x	x
5.99	Toluene, o-chloro	39, 63, 91, **126**	x	x	x	x
6.24	Phenol	39, 66, **94**		x		x
6.25	Aniline	39, 66, **93**	x		x	
6.32	Benzonitrile	50, 76, **103**	x	x	x	x
7.05	Indene	39, 63, 89, 115, **116**	x	x	x	x
7.26	m-tolunitrile	39, 63, 90, **117**	x	x		
7.27	m-cresol	51, 77, 90, 107, **108**			x	x
7.30	p-toluidine	39, 53, 77, 89, 106, **107**	x	x		
7.36	m-toluidine	39, 53, 77, 89, 106, **107**			x	x
8.46	Quinoline	51, 63, 76, 102, **129**	x	x	x	x
8.51	Naphthalene	51, 63, 102, **128**	x	x	x	x
8.59	m-Chloroaniline	39, 65, 92, **127**	x	x		
9.09	3-Chloro-4-methylbenzonitrile	39, 63, 89, 116, **151**	x	x		
9.36	Phenol, 3-chloro-4-methyl	39, 51, 77, 107, **142**	x	x		
9.38	Phenol, 4-chloro-3-methyl	39, 51, 77, 107, **142**			x	x
9.49	p-Toluidine, 3-chloro-	51, 77, 106, 140, **141**	x	x		

9.56	m-Toluidine, 4-chloro	51, 77, <u>106</u>, 140, **141**			x	x
9.67	Phthalic anhydride	50, 76, <u>104</u>, **148**		x		x
9.95	1H-Indene-1,3(2H)-dione	50, 76, 104, **<u>146</u>**		x		
10.52	Similar to 4-Cyanocinnoline	50, 74, 100, <u>127</u>, **155**	x	x		
10.90	Phthalimide	50, 76, 104, **<u>147</u>**		x		x
11.29	2,4-di-tert-Butylphenol	57, 163, <u>191</u>, **206**	x			
11.47	β-Naphthol	89, 115, **<u>144</u>**	x	x	x	x
11.50	Similar to 4-quinolinecarboxaldehyde	51, 75, 101, 129, **<u>157</u>**	x	x		
11.69	Unidentified	114, 140, <u>169</u>	x	x		
11.70	Similar to 2-naphthalenamine	71, 115, **<u>143</u>**			x	x
12.48	Unidentified	114, 154, <u>183</u>	x	x		
13.26	2-Naphthalenol, 1-amino	51, 77, 103, 130, **<u>159</u>**	x	x	x	x
15.76	Unidentified	189, 219, <u>234</u>			x	x
16.16	Unidentified	94, 189, <u>218</u>	x	x		
17.05	Similar to 2H-phenanthro[9,10-b]pyran	101, 202, **<u>232</u>**			x	x
17.13	Unidentified	202, <u>232</u>	x	x		
17.19	Unidentified	115, 205, <u>268</u>			x	x
17.50	Unidentified	189, 202, 218, 233, <u>268</u>	x	x	x	x
17.72	Unidentified	94, 189, <u>252</u>	x	x	x	x
17.83	Unidentified	76, 104, 192, 227, 236, <u>271</u>		x		
17.88	Unidentified	114, <u>245</u>	x	x	x	x
18.67	Unidentified	122, <u>244</u>, 266	x	x		
18.75	9-Chloro-5,6-dihydronaphthol[1,2-c]cinnoline	101, 202, 231, **<u>266</u>**			x	x
18.77	8-Chloro-5,6-dihydronaphthol[1,2-c]cinnoline	101, 202, 231, **<u>266</u>**	x	x		
19.59	Unidentified	115, 127, 241, <u>270</u>			x	x
20.22	Unidentified	121, 243, <u>278</u>	x	x		
21.20	Unidentified	119, 239, <u>268</u>			x	x
21.85	Unidentified	200, 231, <u>330</u>			x	x

Table S5. Main volatile organic compounds detected in the red PS lid 3 (characteristic ions in mass spectra: molecular weight, M_w, in bold and base peak underlined). Additives are marked in *italic*.

Peak number	Compound	*m/z*
1	Toluene	39, 65, 89, <u>91</u>, **92**
2	Ethylbenzene	39, 51, 65, 77, <u>91</u>, **106**
3	Styrene	39, 51, 63, 78, **<u>104</u>**
4	Isopropylbenzene	39, 51, 79, 91, <u>105</u>, **120**
5	Allylbenzene	39, 51, 65, 78, 91, 103, <u>117</u>, **118**
6	α-Methylstyrene	39, 51, 78, 91, 103, 117, **<u>118</u>**

7	3-Butene-1,3-diyldibenzene (styrene dimer)	39, 51, 65, 77, 91, 104, 115, 130, 193, **208**
8	*Drometrizol*	*39, 51, 66, 78, 93, 154, 168, 196, 225*
9	*Palmitic acid butyl ester*	*29, 43, 56, 73, 97, 129, 185, 239, 257,* ***312***
10	*Stearic acid butyl ester*	*29, 43, 56, 73, 97, 129, 185, 241, 267, 285,* ***340***
11	5-Hexene-1,3,5-triyltribenzene (styrene trimer)	65, 77, 91, 117, 194, 207, **312**
12	Isomers of styrene trimer	65, 77, 91, 129, 207, **312**

References

[1] Angelin, E. M.; França de Sá, S.; Picollo, M.; Nevin, A.; Callapez, M. E.; Melo, M. J. The identification of synthetic organic red pigments in historical plastics: Developing an in situ analytical protocol based on Raman microscopy. *J Raman Spectrosc*. **2021**, *52*, 145-158. DOI: **10.1002/jrs.5985**

[2] Hunger, K.; Schmidt M. U. *Industrial Organic Pigments: Production, Crystal Structures, Properties, Applications*, 4th ed.; Wiley-VCH: Weinheim, Germany, 2018.
